# Effectiveness of an Executive Function Training in Italian Preschool Educational Services and Far Transfer Effects to Pre-academic Skills

**DOI:** 10.3389/fpsyg.2019.02053

**Published:** 2019-09-10

**Authors:** Laura Traverso, Paola Viterbori, Maria Carmen Usai

**Affiliations:** Department of Education Sciences, University of Genoa, Genoa, Italy

**Keywords:** executive function, training, pre-academic skills, preschool, intervention

## Abstract

In this study we examine the effectiveness and far transfer effects of a training that was found to be effective in promoting Executive Function (EF) in a sample of 5-year-old children (Traverso et al., 2015). By contrast with Traverso et al. (2015), the intervention was administered by regular teachers to verify its ecological validity. Far transfer was assessed by evaluating the training effects on pre-academic skills. 126 children attending the last year of Italian preschool educational services took part in the study (mainly 5-year-old children). Pre- and post-test assessments were conducted using a large EF and pre-academic skill task battery. The results indicate that the experimental group outperformed the control group in an interference suppression composite score. Moreover, significant far transfer effects to pre-academic skills in literacy domain were found. In addition, we found that the improvement in the pre academic skills (in both literacy and math domains) was mediated by the improvement in the interference suppression score. The results suggest the possibility that this intervention, which may be easily implemented in the context of educational services, can promote EF during the preschool period before entry to primary school.

## Introduction

Executive function (EF) refers to a set of self-regulatory cognitive processes that underlie goal-directed behavior and support individuals faced with new or complex situations (Miyake and Friedman, 2012). In recent years, there has been a considerable interest in the early development of EF, particularly because EF measured in early childhood is a significant predictor of several developmental outcomes, including school readiness (Shaul and Schwartz, 2014) and academic achievement (Best et al., 2011). Consequently, promoting EF may constitute a useful strategy to reduce the neurocognitive disparities among children before school entry and to increase the likelihood of positive developmental outcomes (Blair and Raver, 2015). Recently, promising results have been reported in training studies fostering EF (Diamond and Lee, 2011), nevertheless, some open questions emerged from recent reviews including what are the best methods of improving EF and whether training benefits transfer to other domains (Willis and Schaie, 2009; Jolles and Crone, 2012; Melby-Lervåg and Hulme, 2013; Redick et al., 2015; Diamond and Ling, 2016).

Preschool training includes diverse types of training that differ in duration (long- vs. short-term intervention), setting (individual vs. group intervention), and materials. Training that is delivered by teachers allows to extend the opportunity to train EF before starting primary school to a large sample of children. Nevertheless, not all the available trainings are feasible for all the educational services, such as low resource contexts. Moreover, only a few studies investigated whether benefits in EF, attained with short-term preschool training, transfer to pre academic achievement and the results of these studies are mixed.

The current study was designed to ascertain the effectiveness and the far transfer effects of a short-term school-based intervention that was found to be successful in promoting EFs when a trained psychologist administered it to 5-year-old children (Traverso et al., 2015). Specifically, effectiveness was investigated by verifying the training efficacy when regular teachers of preschool services administered the training in a real-world condition; far transfer was investigated by verifying the effect of EF training on pre-academic skills.

### Preschool Executive Function Development

The preschool years are considered a crucial period in the development of EF during which a significant increase of performance in tasks supposed to assess different EF abilities takes place (see, for example, Garon et al., 2008; Best and Miller, 2010). During the preschool years, besides a quantitative change in EF, a reorganization and progressive identification of different EF skills occurs; specifically, a two-factor structure, in which inhibition and working memory (WM) are distinct but interrelated factors, emerged between 4 and 6 years of age (Miller et al., 2012; Lee et al., 2013; Usai et al., 2014; Monnette et al., 2015; but see Willoughby et al., 2012).

Individual differences in EF reflect substantial genetic contributions at the level of latent variables (Friedman et al., 2008), nevertheless, in recent research it has been highlighted that EF is sensitive to early experience (Kraybill and Bell, 2013; Müller et al., 2013; Raver et al., 2013; Cuevas et al., 2014). Given the prolonged development of EF, it seems plausible that the environment can affect children’s EF, especially when considering environmental factors to which children are extensively exposed to. For example, evidence suggested that factors such as socio-economic status, parenting behaviors and responsive parenting affect the development of EF (Noble et al., 2005, 2007; Farah et al., 2006; Rhoades et al., 2011; Fay-Stammbach et al., 2014). The malleability of EF in response to environmental conditions suggested the possibility of enhancing EF by means of specific stimuli, such as EF training, provided to children in familiar contexts.

### Preschool Executive Function Training

In recent years, EF training has received considerable attention (see Diamond and Ling, 2016, for a review) and diverse types of training have been developed and tested even for preschool children.

Some studies investigated the efficacy of short-term training, consisting of individualized computer training sessions to be carried out over periods ranging from 1 week to 1 month and delivered by researchers in lab (Rueda et al., 2005, 2012; Thorell et al., 2009; Bergman Nutley et al., 2011; Blakey and Carroll, 2015). This approach is based on the idea that EF skills can be enhanced with repeated practice sessions of specific EF tasks; consequently, the effects of this kind of training is generally highly specific (Owen et al., 2010). Positive results were observed in short-term computer training although not in all the EF components that were assessed, in particular positive effects were more often shown in WM tasks than in inhibition tasks (Thorell et al., 2009). Concerning transfer effects on academic achievement, in the study by Blakey and Carroll (2015), transfer effects on math were observed, even though math ability was assessed only at follow up.

Other studies focused on long-term programs, generally group-based interventions that correspond to a school curriculum and are provided in educational services over the entire duration of preschool or during the year before the beginning of primary school (e.g., Bierman et al., 2008a, b; Raver et al., 2011). These teacher-led interventions are mainly designed to improve different aspects of children’s school readiness (for a review see Bierman and Torres, 2016). In a series of studies, Raver et al. (2008, 2009, 2011) evaluated the efficacy of the Chicago School Readiness Project (CSRP) that was effective providing teachers with better classroom management strategies and had the expected impact on the quality of teacher-child interactions (Raver et al., 2008), on children’s aggressive behavior (Raver et al., 2009), on pre-academic skills and on inhibitory control (Raver et al., 2011). Similar results were found for the Head Start REDI program (Bierman et al., 2008b) that showed an impact on children’s EF measures (Bierman et al., 2008b). Another example is the Tools of the Mind Program (Bodrova and Leong, 2007) specifically designed to promote the development of self-regulation skills and that was found to be effective in promoting EF (Diamond et al., 2007; Blair and Raver, 2014). These long-term interventions require extensive teacher training and materials for implementation and are comprehensive in nature, in the sense that are aimed at improving several components of school-readiness, such as self-regulation, social skills, early math, and literacy. The rationale for these interventions is that EF skills can be enhanced in early educational settings by improving the quality of teacher-child interactions and providing supportive educational contexts (Bernier et al., 2012).

As Dias and Seabra (2015) pointed out, it must be noted that these types of training are not suitable for all contexts. For example, some schools may lack key resources to provide computer training or educational interventions that require high-trained personnel. Consequently, both efficacy and effectiveness should be evaluated in order to assess, in the first place, whether a given intervention works under controlled circumstances and then under “real word” conditions and practice (Singal et al., 2014).

To our knowledge, the efficacy of short-term EF training delivered in educational services was investigated in few studies (Röthlisberger et al., 2011; Tominey and McClelland, 2011; Dias and Seabra, 2015; Schmitt et al., 2015; Traverso et al., 2015; Duncan et al., 2018), and even fewer investigated the training effectiveness, that is the training effects on EF when regular teachers administered the training in real-world conditions (Dias and Seabra, 2015; Duncan et al., 2018); finally, only in some short-term training studies, transfer effects on academic performance were examined (Tominey and McClelland, 2011; Schmitt et al., 2015; Duncan et al., 2018). Tominey and McClelland (2011) developed a classroom-based, early childhood intervention that consisted of circle time games implemented in 16 sessions mainly aimed at enhancing behavioral inhibition. *Post hoc* analyses revealed significant effects of the intervention on children with low inhibition scores at pre-test. A second study with children from low-income families showed that the intervention was effective in enhancing self-regulation for the full sample and math skills for English language learners (Schmitt et al., 2015). In both cases, the intervention was administered by the researchers in preschool classrooms. However, in a more recent study (Duncan et al., 2018), the effectiveness of the intervention in improving self-regulation was also demonstrated when it was delivered by teachers as part of an existing kindergarten readiness summer program. No significant effects on early math or literacy skills were found at the end of the program, even though children who took part in the experimental sample showed improved growth in math and literacy during the kindergarten transition period compared with an independent longitudinal sample. However, the results of this study were only partially obtained by a randomized design.

### Executive Function and Pre-academic Skills

Pre-academic skills represent the knowledge a child acquires during the preschool years and include domain-specific precursors of later academic achievement, such as phonological awareness, rapid naming, number recognition, magnitude understanding. These skills are highly predictive of subsequent academic achievement (for a meta-analysis, see La Paro and Pianta, 2000) and contribute to young children’s school readiness (Willoughby et al., 2017). Even though individual differences in preschool EF were consistently found to predict long term math and literacy achievement (Bull et al., 2011; Clark et al., 2013; Miller et al., 2013; Viterbori et al., 2015; De Franchis et al., 2017; Usai et al., 2018), less is known about the predictive associations between EF skills and pre-academic skills. Given that EFs are a set of abilities that support the individual when faced with novel situations, it is plausible that EF influences the acquisition of new abilities or the management of complex cognitive tasks, such as those typical of early reading or writing skills (Blair and Raver, 2015). Nevertheless, as pointed out by Jacob and Parkinson (2015), to date only few studies explored the nature of the association between EF and achievement, with randomized control trial, in preschool age.

### The Current Study

The current study used a randomized design to investigate the effectiveness of a short-term EF preschool training that was previously found to be effective in enhancing EFs when administered by a trained psychologist external to educational service personnel (Traverso et al., 2015); moreover, we aimed to investigate the far transfer effects to pre academic skills.

Specifically, concerning the first aim, whereas efficacy studies, such as Traverso et al. (2015) study, maximizes the likelihood of observing an intervention effect if one exists, effectiveness studies evaluate training under conditions that more closely approach real-world conditions (Singal et al., 2014). In Traverso et al. (2015), the training showed a significant effect on most EF measures, after controlling for pre-test scores. Specifically, the children who took part in the training performed better than the control group in tasks that required delaying a gratification (i.e., Delay Task, adapted from Kochanska et al., 1996), controlling a prepotent behavioral response (i.e., the Circle Drawing Task and Preschool Matching Familiar Figure Task), managing interference (i.e., Flanker Task) and high cognitive conflict (i.e., the Dots task), and in tasks assessing WM (i.e., Backward Word Span and Keep Track). The effect size (Cohen’s *d*) ranged from 0.35 to 0.70 and it was from medium (>0.50) to large (0.80) for the majority of the tasks. In the current study, we were interested in verifying whether the EF gains obtained in the study by Traverso et al. (2015) could be found also when regular teachers, minimally trained, administered the training during the daily school schedule in real-world conditions. As pointed out by Singal et al. (2014), in effectiveness studies, providers may adopt less-standardized protocols and target a more heterogeneous children population. Indeed, differently from Traverso et al. (2015), we decided to include in the study also the children with special needs who in Italy attend regular classes (see Zanobini et al., 2017).

In addition, as regards the second aim, differently from the previous study, the present one was designed to assess whether EF training effects could transfer to pre-academic skills. Specifically, we were interested in verifying whether an increase in EF skills could enable children to benefit more from learning opportunities and consequently enhance their pre-academic skills, even without an intervention directed at these skills. To date the far transfer of short-term EF training delivered by teachers to pre-academic skills in preschoolers has been rarely investigated (Duncan et al., 2018). In addition, as suggested by Bierman and Torres (2016), we employed an analytical approach that allows to control for dependencies associated with influences due to the belonging to different classes.

Similarly, to the intervention used in Duncan et al. (2018), the training involved low-cost and easily available materials (e.g., colored markers, pens, and pencils) and lasted approximately 1 month. Moreover, the activities were designed to be included in the standard preschool curriculum, which in Italy emphasizes learning through play and adopt a small-group approach. Indeed, we were interested in comparing the training condition with usual practice. Differently from Duncan et al. (2018) whose training activities were designed to primarily practice inhibitory control, our training focused on both inhibitory control and WM, and we used a large battery of EF tasks at pre- and post-test.

To summarize, we examined two research questions: (1) whether a short-term training designed to foster EF in children of 5 years of age showed ecological validity, being effective in promoting executive skills when administered by regular teachers with all the children; (2) whether the training produced far transfer effects on pre-academic skills.

## Materials and Methods

### Participants

A total of 137 5-year-old children attending the last year of seven preschool educational services participated in the study. Public preschools in Italy enroll children from 3 to 5 and offer a pre-primary curriculum that promotes social skills, autonomy, and learning. Even though attendance is non-compulsory, more than 95% of target children attend preschool before starting primary school at the age of six. During the last year of preschool, which corresponds to kindergarten level in the US, particular attention is paid to school-readiness and acquisition of pre-academic skills, such as early reading and writing skills, phonological awareness, and number sense.

1The selected preschools serve the same urban area of two large cities in a northern Italian region. In agreement with the school principals and teachers, the study was presented to the parents of the children attending the last year of preschool; the parents who agreed to allow their children to participate filled in the parental informed consent form. This study was carried out in accordance with the recommendations of the Ethical Code of the Italian National Council of Psychologists and the Ethical guidelines of the Italian Association of Psychology.

Eleven children were excluded from the initial sample because they did not take part in the assessment at pre- or post-test evaluation. We were therefore interested in verifying whether the training was effective also when administered in regular classes that may include children with special educational needs. In particular, 21 children with special needs participated, specifically nine children with atypical developmental paths (i.e., born pre term, presenting language delays, or with attention difficulties) (eight in the control group), one child in the care of social services (one in the control group); two minority language children with limited proficiency in Italian (two in the control group), nine children with a score under the 10th percentile in the Raven’s colored progressive matrices (three in the control group). Children with special needs were not evenly distributed between the experimental and the control group, since group allocation was according to class.

The final sample included 126 children between the ages of 52 and 78 months (M_age_ = 65.4 months; SD = 4.31; 44% females) who were attending the last year of preschool services before starting primary school: 57 children were in the control group (M_age_ = 66.1; SD = 4.29; 47% females) and 69 children were in the experimental group (M_age_ = 64.9; SD = 4.28; 42% females).

In determining the sample size we referred to previous studies in which short-term training were assessed (i.e., Tominey and McClelland, 2011; Blakey and Carroll, 2015; Dias and Seabra, 2015; Traverso et al., 2015). The children attended 7 preschools and were grouped in 13 classes. Preschools were randomly assigned to the control condition (four preschools, four classes) and to the experimental condition (three preschools, nine classes), in order to have a similar sample size and to ensure that teachers of the control group of children were unaware of the intervention stimuli, and that teachers of the experimental group of children did not transfer the training activities to the control group. We do not include an active control group because children of both the control and experimental group spend the same time with teachers in similar settings, and are normally involved in small-group educational workshops. Specifically, in Italy, the preschool classrooms comprise from 18 to 26 children between 3 and 5 years of age. In each classroom there are two teachers with some hours of co-teaching, during which they usually organize small group activities for children of the same age.

### The Training

The training program was the one described in Traverso et al. (2015). It included 12 sessions of approximately 30 min that were administered at school three times a week over approximately 1 month.^1^ While in the Traverso et al.’s (2015) study the training was administered to small groups of five children, in the current study the groups ranged from 5 to 8 children. The training aimed to stimulate EF skills through a series of small group game activities that require progressively higher levels of inhibitory control and working memory and require children’s active participation. Each child was given a different role with a specific responsibility (i.e., the director, the referee, the player) – for example, the director was in charge of managing the players’ behavior. During each session, the roles were exchanged. For example, in the second activity, children must help the Magic Frog become better able to inhibit irrelevant information and control its actions. The director has to regulate attention in naming a series of pictures on a paper, and he asks the players to touch the floor or jump according to what they hear and what they have as assigned pictures. The referee must assign a score only if all the players move correctly. All of the training activities were different from the assessment tasks that were administered to the children before and after the intervention.

In order to help children manage the activities, we use a narrative framework that enables young children to connect and remember the activities from one session to the other and to be more focused and motivated, since the activities are included as a part of the story in which they have to help two little goblin friends. Moreover, each session is structured in the same way. First, an introductory activity helps children to recall the rules they are asked to respect and to bring to mind what happened in the previous session; then, the specific EF activities are presented to children and, in the end children are engaged in a metacognitive activity during which they have to assess their performance and to briefly discuss the strategies they used in managing the activities. We provided concrete aids to help the children develop and practice self-regulation strategies through concrete experiences with physical materials. Finally, the adult that administers the activities is asked to pay special attention to support the children’s self-esteem and well-being during the activities, and to praise the children for their efforts during and at the end of each session.

By contrast with Traverso et al. (2015), in which the training was carried out by a trained psychologist, in this study regular teachers administered the training to all the pupils of their class. A training manual (see Supplementary Material) and a 12-h in-service course were provided to the teachers that participated in the study. Specifically, the training manual included a general description of the training’s aims, the description of the activities, the instructions to administer the activities, and some printed materials to be used during the training. The course took place concurrently with the training and included six 2-h-sessions. The first session focused on EF and its role in early education, and provided a description of the general characteristics of the training. In the second session, the first three training activities were presented to the teachers and in addition, teachers were given all the instructions to prepare the materials. In the following three sessions, the other activities were presented (three at a time) and teachers were encouraged to discuss their experience with the administration of the previous three activities; in addition, the adherence to the program was assessed and discussed with the teachers. Finally, in the last session, teachers were encouraged to discuss their global experience with the training.

### Assessment Procedure

The control and the experimental groups were assessed before and after the training. Children were tested individually in a quiet room in three separate sessions, each lasting approximately 20 min. Evaluations were made within 2 weeks before and after training. The tasks were presented in a fixed order (Table 1). A fixed order is a standard practice in individual differences research (see Carlson and Moses, 2001). All the tasks described in the following section were administered twice (i.e., pre- and post-training), with the exception of the Coloured Progressive Matrices Test (CPM, Raven, 1954), which was used as a control measure concerning cognitive functioning of the two groups at pre-test. In both pre- and post-training conditions, trained psychologists, blind to the children’s group assignment, tested the children individually.

**TABLE 1:** Summary of the assessment battery: the order of tasks for each session and the variable labels used in each task to assess cognitive abilities, EF, and pre-academic skills are reported.

	**Task order for each sessions**	**Variables (score range)**	**To assess**
1° Session	Coloured progressive matrices Backward word span Preschool matching figure task Keep track	CPM, sum of correct item (0–36) Backward span, span level (1–9) Matching, errors (0–56) Keep track, sum of correct item (0–9)	Intelligence WM Response inhibition WM
2° Session	Fish flanker task Circle drawing task Dots task	Flanker, accuracy (0–16) Circle drawing, proportion of slow down Dots, accuracy (0–20)	Interference suppression Response inhibition Interference suppression
3° Session	Digit comparison task Digit-dots correspondence Rapid automatic naming Identifying the rhymes Syllable fusion Writing task	Digit comparison, accuracy (0–11) Digit correspondence, accuracy (0–9) Rapid naming, errors (0–no limit) Rhymes, accuracy (0–19) Syllable, accuracy (0–18) Writing task, accuracy (0–6)	Early math Early math Rapid naming Phoneme awareness Phoneme awareness Early writing skills

### Measures

#### Fluid Intelligence

The Coloured Progressive Matrices Test (Raven, 1954) was administered to measure fluid intelligence and was used as a control. It is a multiple choice test of abstract reasoning in which the child is required to complete a geometrical figure by choosing the missing piece among six possible drawings; the patterns progressively increase in difficulty during the 36 items presented (CPM, expected range 0–36).

#### Executive Function Battery

To assess EF, the following tasks were administered.

##### Circle drawing task

This task (Usai et al., 2017, adapted from Bachorowski and Newman, 1985) was used to evaluate response inhibition, specifically the motor inhibition of an on-going response (Geurts et al., 2005; Marzocchi et al., 2008; Usai et al., 2014). The child must trace with his finger over a 17 cm diameter circle from a starting point to an ending point. The task is administered twice. On the first administration, neutral instructions (“trace the circle”) were given, and on the second administration inhibition instructions were given (“trace the circle again but this time as slowly as you can”). Larger time differences indicate better inhibition (slowing down) on the part of the participant in their continuous tracing response. Time in seconds was recorded for each trial. Scores were calculated as the slowdown relative to the total time using the following formula: T2-T1/T2 + T1, where T1 and T2 were the times recorded for the first and second trials, respectively (Circle drawing, expected range negative to positive values-no limit). The test–retest reliability coefficient was calculated on a sample of 43 5-year-olds, who had been assessed twice in a previous study by Traverso et al. (2015). The Pearson correlation coefficient was 0.57.

##### Preschool matching familiar figure task

This task (Traverso et al., 2016; Usai et al., 2017) measures the child’s ability to restrain impulsive responses and to compare the target with all of the pictures by shifting attention from the target to each alternative. The child is asked to select the figure that is identical to the target picture at the top of the page from among different alternatives. In the format adapted for kindergartners, this task involves five alternatives and comprises 14 items. The number of errors was recorded (Matching, expected range 0–56). The Cronbach’s alpha calculated in a sample of 174 children (M_age_ = 60.04) was 0.67 (Traverso et al., 2016).

##### Fish flanker task

The Flanker task (Usai et al., 2017, adapted from Ridderinkhof and van der Molen, 1995) is a well-known paradigm that is used to evaluate the ability to inhibit irrelevant interfering stimuli (Eriksen and Eriksen, 1974). The child is required to respond to a left or right oriented fish that is presented at the center of the computer screen by pressing a left or right response button. Two other fish facing the same (congruent condition, 16 items) or opposite direction (incongruent condition, 16 items) flank the target fish. After a brief training session consisting of four items (two of each condition), thirty-two items are randomly presented (16 items per condition, half left and half right). A warning cross (500 ms in duration) preceded the stimulus. After the response, the screen turned blank for 500 ms. Accuracy in the incongruent condition (Flanker, expected range 0–16) was recorded. Test–retest reliability (Pearsons’ *r*) calculated in a sample of 43 typically developing children (age range 62–75 months, M_age_ = 68.60; SD = 3.5) was 0.42 (Usai et al., 2017).

##### Dots task

This task (Usai et al., 2017 adapted from Diamond et al., 2007) is a high cognitive conflict task that requires both inhibition and WM (Diamond et al., 2007). In this task, the child has to shift between rules according to the stimulus presented (see Diamond et al., 2007; Diamond and Lee, 2011). A heart or a flower appears on the right or left of a computer screen. The child is told that he must press on the same side as the heart but on the side opposite the flower, which requires inhibiting the tendency to respond on the side where the stimulus appeared. After a brief training session with heart and flower items, the test began, and hearts and flowers were intermixed in the test. The sum of the correct responses (Dots, expected range 0–20) was recorded. Test–retest reliability (Pearson’s *r*) calculated in a sample of 43 typically developing children (age range 62–75 months, M_*age*_ = 68.60; SD = 3.5) for accuracy was 0.62 (Usai et al., 2017).

##### Backward word span

This task is a traditional WM task (Carlson, 2005; Alloway et al., 2006). This task requires the child to recall a sequence of spoken words in reverse order. Words were presented approximately once per second. After an illustration trial, the test begins with three trials of two words. The number of words increments by one every three trials until three lists are recalled incorrectly. The maximum list length at which two sequences were correctly recalled was scored (Backward span, expected range 1–9).

##### Keep track

The Keep track task (Usai et al., 2017 adapted by Van der Ven et al., 2011) is a WM task that is suitable for assessing updating ability in both adults (Miyake et al., 2000) and children (Van der Sluis et al., 2007; Van der Ven et al., 2011). The child was shown pictures, each of which belonged to one of the following five categories: animals (dog, cat, fish, bird), sky (sun, moon, stars, cloud), fruit (strawberry, grape, pear, apple), vehicles (train, bicycle, motorbike, car), and clothes (socks, skirt, t-shirt, shoes). Before each trial, the child was asked to pay special attention to one (first three trials) or two designated categories (last three trials). The pictures were shown in series of six. During the presentation of each series, the child had to name each picture. At the end, the child had to recall the last item in each designated category, which required managing the interference caused by the other named pictures. The number of designated categories increased from one (in the first three series) to two (in the last three series). During the picture presentation, small pictures symbolizing the categories to be remembered were shown at the bottom of the screen to serve as a reminder. One point was given for each correct response, and 0.5 points were given if the child was not able to recall the item and asked to see all the pictures in the requested category again (Keep track, expected range 0–9). Test–retest reliability (Pearson’s *r*) calculated in this sample (typically developing children of the control group) was 0.544.

#### The Pre-academic Skills Battery

To assess pre-academic skills, the following tasks were administered.

##### Early math skills

We administered two subtests of the Numerical Intelligence Battery (BIN, Molin et al., 2007), a standardized battery for the assessment of numerical competence in preschool children. In the digit comparison task, children have to choose the larger of two Arabic digits and receive one point for each correct response. The task is composed of eleven trials with digits ranging from 1 to 9 (Digit comparison, expected range 0–11). In the digit-dots correspondence, the children have to match the digit presented with the corresponding set of dots among three visually presented sets. The task is composed of nine trials and children receive one point for each correct response (Digit correspondence, expected range 0–9).

##### Early literacy skills

We administered two subtests of the PAC-SI (Scalisi et al., 2003) and one of the CMF (Marotta et al., 2008), that are two standardized batteries for the assessment of pre-academic skills in preschool children. In the Rapid automatic naming task (PAC-SI), children must name a series of different objects, which are in different sequences and divided into six rows, as quickly as possible and in order from left to right. Errors (Rapid naming, expected range 0–no limit) were measured. In the Identifying the rhymes task (PAC-SI), children are shown three pictures have to name the pictures aloud and identify the word that does not rhyme with the others. The test includes 19 items. The score (Rhymes) is the number of words correctly identified by the children (expected range 0–19). In the Syllable fusion test (CMF), after listening children had to put syllables into one word and pronounce it. The test includes 18 items (six words with three, four and five syllables). The score (Syllable) is the number of correct words repeated by the children (expected range 0–18).

In addition, children were asked to perform a spontaneous handwriting task (Writing task), in which they had to write the name of four different pictures (a dog, a table, a sun, and an elephant). Based on Ferreiro and Teberosky’s (1982) model of writing acquisition, children’s performance was scored as follows: writing via drawing or scribbling (1 point), writing via making letters like forms (2 points), writing via reproducing at least one correct letter (3 points), writing via reproducing well-learned units (4 points), writing via invented spelling (5 points) and writing via conventional spelling (6 points). A score ranging from 1 to 6 was assigned to each of the four figures. The final score was given by the mean of the scores obtained in each of the four pictures. Two judges coded the children’s performance independently. The correlations between the two judges indicated adequate coding reliability (pre-test, *r* = 0.986; post-test, *r* = 0.993).

### Statistical Analyses

Descriptive analyses and pre-test comparisons with Student’s *T*-test and Chi-square were conducted to investigate differences between the control and the experimental groups at baseline in relation to EF task scores, pre-academic skill tasks performance, age, fluid intelligence, level of mother’s education, and gender distribution. Zero-order (Pearson) correlations among measures were calculated. Given that EFs are usually low associated (Willoughby et al., 2016), in order to improve precision of measurement, Willoughby et al. (2017) suggest to administer multiple tasks and aggregating performance across these tasks (formative indices). Therefore, to perform the subsequent analyses, the pre- and post-test scores were transformed into *z*-scores. Each *z*-score for the post-test was calculated using the mean and the standard deviation derived from the pre-test phase, thus obtaining a *z*-score gain. Based on the literature, which suggests that two components of inhibition emerge as separate at this age (Gandolfi et al., 2014; Traverso et al., 2018), two inhibitory composite scores were calculated as the mean of the *z* scores: a response inhibition score with the Circle drawing score and the Preschool matching familiar figure score (multiplied by −1), and an interference suppression score with the Fish flanker and the Dots tasks. Moreover, a composite score for WM abilities was obtained with the Backward word span and the Keep track tasks. The Math and the Literacy composite scores included the Digit comparison and the Digit correspondence scores, and the Rhymes, the Syllable fusion, and the Rapid automatic naming (multiplied by −1) scores, respectively. The composite scores for each participant were calculated when both or two out three values of the original variables were present. Zero-order (Pearson) correlations among composite scores were calculated. Then, for each child the three EF composite scores, the Literacy and the Math composite scores and the spontaneous handwriting score (Writing score) were submitted to a series of repeated measures linear mixed model (LMM) analyses using General Analyses for Linear Model (GAMLj) in a Jamovi package (The Jamovi Project, 2018). The LMM enables taking into the account the dependency among the measures within clusters; in this case, we can consider the dependency effects in the models to be due to the participants’ characteristics and the class attended. Moreover, the LMM enables efficient handling of missing values because it does not employ a listwise procedure. Specifically, mixed models uses maximum likelihood, which handles the missing data. In each LMM, the EF composite scores, the Literacy and Math composite scores and the Writing score were modeled as fixed factors; participant and class intercepts were considered as random factors and age as covariate. To investigate the training efficacy the interaction between Time of assessment (pre- and post-test) and Group was included. This analysis was used to test our hypotheses for each dependent variable. To verify the relative magnitude of the training, the *d* effect size was calculated using Morris (2008) effect size formula for mean differences of groups with unequal sample size within a pre-post-control design.

In order to investigate the relationship between experimental condition, EF and pre-academic skills a mediation analysis was executed with the Bootstrapping method (Preacher and Hayes, 2008), implemented in the MedMod package of Jamovi software. This type of analysis enables verifying whether the relationship between two variables (group condition and pre-academic skill scores) depends on another variable (EF score).

## Results

### Baseline Level

Descriptive statistics for all the tasks for both the control and the experimental groups at pre- and post-test are reported in Table 2. A high percentage of missing values was observed in the Writing task (children refused to perform the task) and in the Dots and Flanker task (due to a computer problem which caused data loss).

**TABLE 2:** Descriptive statistics for the experimental and the control group in the pre- and in the post-test phase.

		**Pre-test**			**Post-test**		
**Tasks’ variables**	**Groups**	***n***	***M***	***SD***	***n***	***M***	***SD***
Circle drawing	Control	54	0.37	0.49	55	0.35	0.48
	Experimental	69	0.36	0.48	68	0.43	0.50
Matching errors	Control	57	11.86	6.93	57	9.56	5.95
	Experimental	69	13.01	5.70	68	9.47	5.37
Flanker accuracy	Control	55	11.86	4.52	52	13.11	4.19
	Experimental	54	12.60	4.31	67	15.20	1.74
Dots accuracy	Control	55	14.00	4.03	56	15.00	4.30
	Experimental	57	13.39	4.15	67	16.37	4.00
Backward span	Control	56	1.95	0.80	56	2.21	0.65
	Experimental	69	1.93	0.80	69	2.22	0.66
Keep track	Control	57	3.72	2.33	57	5.08	2.39
	Experimental	69	3.38	2.04	69	5.13	1.90
Digit comparison	Control	57	8.39	2.56	55	9.26	3.13
	Experimental	69	8.75	2.79	66	9.82	1.82
Digit correspondence	Control	57	6.90	2.15	57	6.75	2.81
	Experimental	69	6.33	2.63	69	7.15	2.40
Syllable	Control	57	11.98	5.29	57	13.72	5.95
	Experimental	69	11.59	5.00	69	14.12	5.41
Rhymes	Control	57	7.98	4.31	57	8.70	5.05
	Experimental	69	8.33	4.21	69	9.49	5.10
Rapid naming	Control	53	0.57	1.03	51	0.98	1.27
	Experimental	67	0.58	1.03	65	0.40	0.08
Writing task	Control	54	2.65	1.58	50	3.02	1.61
	Experimental	63	3.10	1.59	62	3.73	1.73

At pre-test no difference emerged between the two groups in EF and pre-academic skills. Moreover, no difference emerged between the control and the experimental group in the CPM score (control group mean = 16.75, SD = 4.69, experimental group mean = 16.33, SD = 3.85), in the level of mother’s education (school years; control group mean = 12.5, SD = 3.34, experimental group mean = 13.5, SD = 3.56), and in children’s age (all *p*s > 0.05). Zero-order (Pearson) correlations among EF and pre academic skill measures were calculated (Table 3). As expected, the EF task scores were not highly related (Willoughby et al., 2016). Moreover, zero-order (Pearson) correlation among composite scores were calculated (Table 3).

**TABLE 3:** Zero-order correlations among EF and pre academic skills tasks (measures scores) and among composite score.

**Measures scores**												
	**1**	**2**	**3**	**4**	**5**	**6**	**7**	**8**	**9**	**10**	**11**	**12**
Circle	1	0.004	0.133	0.348^∗∗^	0.162	0.054	0.11	0.194^∗^	0.244^∗∗^	0.241^∗∗^	0.046	0.279^∗∗^
Matching		1	−0.327^∗∗^	–0.267^∗∗^	–0.255^∗∗^	–0.271^∗∗^	−0.178^∗^	−0.184^∗^	–0.168	−0.226^∗^	0.113	–0.092
Flanker accuracy			1	0.278^∗∗^	0.320^∗∗^	0.128	0.282^∗∗^	0.252^∗∗^	0.154	0.13	0.102	0.214^∗^
Dots accuracy				1	0.410^∗∗^	0.148	0.257^∗∗^	0.435^∗∗^	0.225^∗^	0.243^∗∗^	–0.149	0.329^∗∗^
Backward span				1	0.175	0.373^∗∗^	0.500^∗∗^	0.327^∗∗^	0.241^∗∗^	–0.153	0.311^∗∗^	
Keep track					1	0.202^∗^	0.284^∗∗^	0.211^∗^	0.092	–0.045	0.072	
Digit comparison						1	0.597^∗∗^	0.289^∗∗^	0.355^∗∗^	–0.042	0.314^∗∗^	
Digit correspondence						1	0.404^∗∗^	0.414^∗∗^	–0.268^∗∗^	0.288^∗∗^		
Syllable									1	0.386^∗∗^	–0.059	0.385^∗∗^
Rhymes										1	–0.071	0.412^∗∗^
Rapid naming										1	–0.117	
Writing task											1	

**Composite scores**												
		**1**		**2**		**3**		**4**		**5**		**6**

Response inhibition		1		0.459^∗∗^		0.360^∗∗^		0.284^∗∗^		0.344^∗∗^		0.317^∗∗^
Interference suppression		1		0.404^∗∗^		0.334^∗∗^		0.216^∗^		0.422^∗∗^		
WM						1		0.250^∗∗^		0.235^∗∗^		0.481^∗∗^
Literacy						1		0.366^∗∗^		0.352^∗∗^		
Writing task						1		0.419^∗∗^			
Math								1			

*^∗^*p* < 0.05; ^∗∗^*p* < 0.01.*

### Training Effects on EF and on Pre-academic Skill Tasks

**FIGURE 1:**
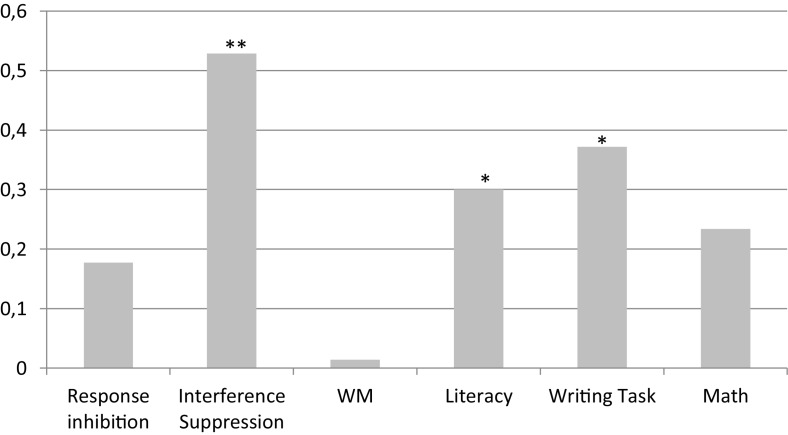
Effect sizes of the gain scores at Time 2. ^∗^*p* < 0.05; ^∗∗^*p* < 0.001.

To test the efficacy of the training a series of repeated measures analyses with the LMM was conducted on the three EF composite scores (response inhibition, interference suppression, and WM), on the Literacy and Math composite scores, and on the Writing score. Variance for the random effect due to participants ranged from 0.44 (for the Response inhibition score) to 0.89 (for the Writing task). Variance for the random effect due to the class attended ranged from 0.01 (for the Math score) to 0.08 (for the Writing task). Considered the aim of this study, only the interaction between Group (experimental and control) and Time (pre- and post-test phases) was considered and the results are shown in Table 4. This interaction was significant for the Interference suppression, the Literacy and the Writing task scores. The inspection of simple effects showed that the experimental group (*B* = 0.303, SE = 0.042, *t* = 7.25, *p* < 0.001) and control group (*B* = 0.125, SE = 0.042, *t* = 2.96, *p* = 0.004) both presented an increase in performance from time 1 to time 2, but this gain was greater for the experimental group. Age does not show significant effects in any model. Effect sizes for the gains obtained at Time 2 are shown in Figure 1.

**TABLE 4:** Composite *z*-scores for the two groups at the pre- and the post-test phase.

		**Pre-test**			**Post-test**						
**Composite *z*-scores**	**Groups**	***n***	***M***	***SD***	***n***	***M***	***SD***	***F***	***p***	***R*^2^_marginal_**	***R*^2^_conditional_**
Response inhibition	Control	54	0.03	0.72	55	0.18	0.70	2.000	0.160	0.037	0.487
	Exp.	69	–0.05	0.70	67	0.30	0.66				
Interference suppression	Control	54	0.00	0.79	52	0.31	0.79	8.391	0.005	0.101	0.708
	Exp.	54	–0.00	0.83	65	0.67	0.58				
WM	Control	56	0.06	0.82	56	0.55	0.77	0.399	0.529	0.119	0.574
	Exp.	69	–0.04	0.72	69	0.54	0.69				
Math	Control	57	0.03	0.84	55	0.21	1.05	2.684	0.104	0.039	0.671
	Exp.	69	–0.02	0.94	66	0.41	0.65				
Literacy	Control	53	0.06	0.68	51	0.22	0.70	4.14	0.044	0.080	0.655
	Exp.	67	0.04	0.59	65	0.42	0.64				
Writing task	Control	54	–0.15	0.99	50	0.13	1.01	4.470	0.037	0.067	0.841
	Exp.	63	0.08	0.64	62	0.52	1.08				

*LMM results for the interaction between group and time controlling for age.*

### Mediation Analysis

**FIGURE 2:**
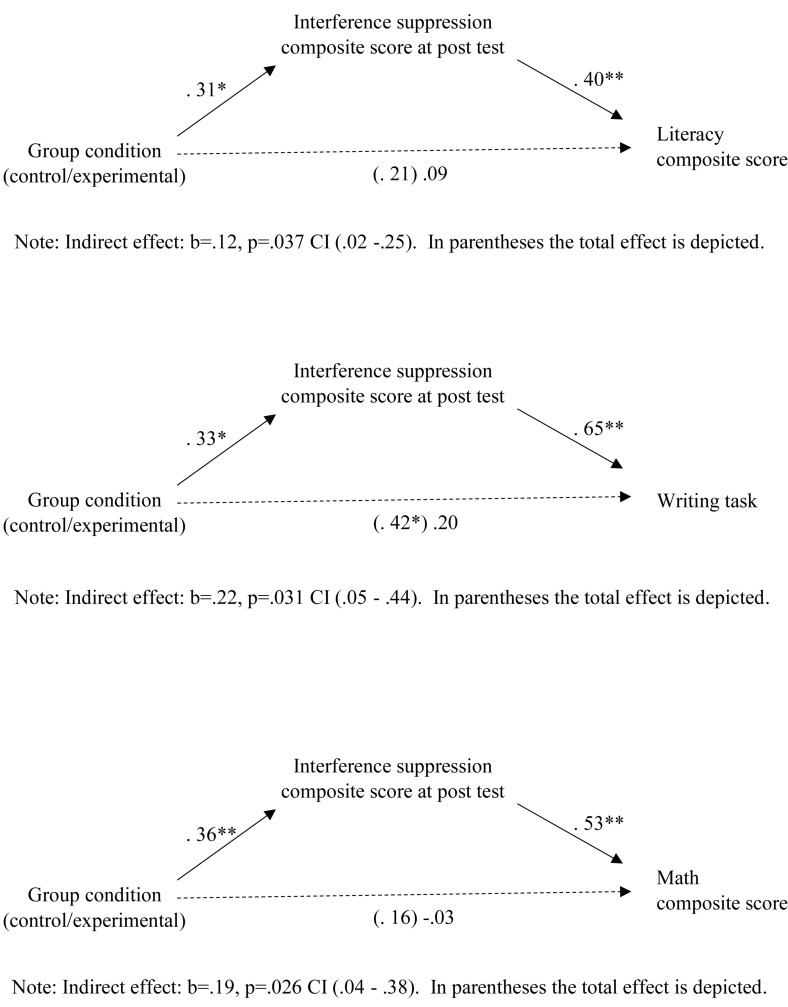
Results of mediation analysis. ^∗^*p* < 0.05; ^∗∗^*p* < 0.001.

In order to investigate the relationship between experimental condition, EF and pre-academic skills a mediation analysis was performed considering only the measures that were improved by the training (interference suppression, literacy, and writing). A full mediation effect was observed when we entered Literacy as a dependent variable, group condition as an independent variable and the interference suppression score as a mediator. A significant effect for the indirect path was found (*Z* = 2.084, *p* = 0.037; 57.5% of the total effect), but not for the direct path (*Z* = 0.847, *p* = 0.397). The group condition predicted the interference suppression composite score, specifically the experimental group showed higher levels of interference suppression compared to the control group in the post-training assessment (β = 0.31, SE = 0.14, *Z* = 2.264, *p* = 0.024). Supportive to our mediation hypothesis, when the interference suppression composite score was entered into the model as a mediator, the effect of group condition on the Literacy score turned non-significant (β = 0.09, SE = 0.11, *Z* = 0.847, *p* = 0.397), whereas the effect of the interference suppression score on the Literacy score was significant (β = 0.40, SE = 0.07, *Z* = 5.462, *p* < 0.001) and also the indirect path from group condition to pre-academic skills through FE improvements was significant, *a × b* = 0.12, Bootstrap 95% CI [0.02,0.25] (Figure 2).

Similarly, when we repeated the analyses entering the Writing task as a dependent variable, a significant effect for the indirect path was found (*Z* = 2.16, *p* = 0.031; 51.6% of the total effect), but not for the direct path (*Z* = 1.03, *p* = 0.302). The experimental group belonging predicted higher levels of interference suppression (β = 0.33, SE = 0.14, *Z* = 2.37, *p* = 0.018). When the interference suppression composite score was entered into the model as mediator, the effect of group condition on the Writing performance turned non-significant (β = 0.20, SE = 0.20, *Z* = 1.03, *p* = 0.302), while the effect of the interference suppression on the Writing score was significant (β = 0.65, SE = 0.12, *Z* = 5.34, *p* < 0.001) and the indirect path from group condition to pre-academic skills through FE improvements was significant, *a × b* = 0.22, Bootstrap 95% CI [0.05,0.44] (Figure 2).

In sum, the experimental group outperformed the control group in the Interference suppression ability represented by the Fish flanker and the Dots task. The experimental group’s improvement in the Literacy and in the Writing task was mediated by interference suppression improvement showing that the training produced significant far transfer effects.

Moreover, although total effect was not significant, according to Hayes (2009), we performed a mediation analysis considering the Math composite score as dependent variable. A partial mediation effect was observed when we entered Math as a dependent variable, group condition as an independent variable and the interference suppression score as a mediator. A significant effect for the indirect path was found (*Z* = 2.232, *p* = 0.026; 86.6% of the total effect), but not for the direct path (*Z* = −0.222, *p* = 0.824). The group condition predicted the interference suppression composite score, specifically the experimental group showed higher levels of interference suppression compared to the control group in the post-training assessment (β = 0.36, SE = 0.13, *Z* = 2.690, *p* = 0.007). Moreover, the effect of the interference suppression score on the Math score was significant (β = 0.53, SE = 0.12, *Z* = 4.598, *p* < 0.001) and also the indirect path from group condition to pre-academic skills through FE improvements was significant, *a × b* = 0.18, Bootstrap 95% CI [0.04,0.38].

## Discussion

### The Training Effectiveness

This study adds to the literature examining the effects of EF training in preschoolers. In particular, the rationale for designing such a study was to test the effectiveness of a short-term intervention that previously proved to be effective in promoting EF (Traverso et al., 2015). Differently from the study by Traverso et al. (2015) in which training efficacy was evaluated when the training was administered in high controlled conditions by a trained psychologist external to the educational service personnel, in the current study, regular teachers, minimally trained, administered the training to all the pupils of their class in real world conditions. Diamond and Ling (2016) suggest that the way in which an activity is presented and conducted may influence the results in terms of gains. Indeed, when the intervention was administered by a trained psychologist who was “committed to it succeeding and believes firmly in its efficacy” (Diamond and Ling, 2016, p. 37), a significant increase in a wide range of EF skills was found (Traverso et al., 2015). The results of the present study indicate that the intervention is still effective in promoting EF abilities even when administered by regular teachers. Specifically, in Traverso et al. (2015) effects were observe in WM, response inhibition and interference suppression tasks, in the current study the intervention still produced an increase in interference suppression abilities with a comparable effect size.

Another important issue concerns the composition of the groups. It should be noted that in Italy children with special needs attend regular classes. Thus, the experimental and the control samples included all the children for whom parents gave their consent to participate, including children with special needs. Even if it was not possible to examine training effects on children with special needs due to the low number of children, results showed that in average the experimental group, in which special needs children were included, outperformed the control group in both EF (interference suppression) and pre-academic skills tasks (literacy domain). This is an important point because these results support the idea that there were no barriers that affect participation of children with special needs, therefore this kind of intervention appears to be suitable for inclusive educational contexts. Moreover, given that inclusive contexts are generally highly challenging, we may assume that this training may be easily administered by teachers in real classes even in educational contexts outside the Italian inclusive system.

The literature on EF interventions recommends the use of intent-to-treat analyses to avoid biases due to intervention drop-out, in addition to a nested design to account for teacher and school influences (e.g., Bierman and Torres, 2016). In this study the dependencies associated with the presence of participants belonging to the same classes were modeled using the LMM that, in addition, enables managing missing data without employing a listwise procedure. The participants’ characteristics and the way in which the intervention was implemented may support the generalization of these results to the population.

The narrower training effects as compared to Traverso et al.’s (2015) study were possibly due to fact that the training was administered by teachers instead of a specialized psychologist; nevertheless, the reasons why the training produced positive results in some but not all the EF tasks must be discussed. Specifically, the training group outperformed the control group in the interference suppression ability represented by the Fish flanker task and the Dots task, while response inhibition and WM were not affected by the training. These results can be explained by considering the types of skills we considered and the developmental trajectories of these skills. The ability to suppress a prepotent but inappropriate response to a stimulus (response inhibition) appears early whereas interference suppression, that is the ability to address conflict or interference from complex and misleading features of a task, develop later (Gandolfi et al., 2014). Considering their later development, interference suppression skills could be possibly characterized by a higher plasticity in the age group examined, thus resulting more sensitive to external stimulations. In fact, according to Cragg (2016), performance enhancement in inhibitory tasks during middle childhood may be explained mainly by the improvement in interference suppression rather than in response inhibition ability. Following this line of reasoning, response inhibition and WM increase may require more extensive effort to produce appreciable changes. The present intervention, which is composed of 12 sessions, may not be sufficient to produce a significant improvement in these abilities. As also suggested by Diamond and Ling (2016) EF gains certainly depend on the amount of time spent practicing, but the optimal amount of practice to produce significant results has not yet been ascertained (Bierman and Torres, 2016). Finally, it should be noted that although the training did not increase the experimental group’s performance in all the tasks, the dissimilarity between the training activities and the tasks adopted in the assessment leads us to assume that we measured real improvements in EF capacity and not a mere task-training effect.

### The Issue of the Far Transfer

This study also examined the far transfer of the training to pre-academic skills. As noted by Bierman and Torres (2016), an issue of great importance for early education and prevention policy is the degree to which the improvement in specific EF tasks extends to learning or behavioral outcomes. Although the predictive relationship between preschool EF and school achievement has been well-established (Viterbori et al., 2015; De Franchis et al., 2017), less is known about the relationship between EF and pre-academic skills and about the possibility of bringing about improvement in pre-academic skills and school readiness through EF training.

For the aim of this study, the question was whether the improvement in interference suppression could promote an enhancement in the level of pre-academic skills.

The results show an improvement in early literacy and in writing skills and suggest the existence of a direct effect of EF on these pre-academic skills. Moreover, our results showed that the training improved the interference suppression composite score, which in turn accounted for Math composite score.

Considering the results of the full mediation in the literacy domain, evidence suggests that early spelling attempts predict subsequent word reading and interventions that improve this ability in the last year of preschool can consequently promote an advantage in reading acquisition (Ouellette and Sénéchal, 2008). Moreover, research suggests that EF skills are strong correlates of young children’s emergent literacy skills in kindergarten (e.g., phonemic awareness and letter knowledge) (Blair and Razza, 2007). In particular, Zhang et al. (2017) found that preschoolers with stronger EF skills achieved higher gains in letter-sound knowledge, which, in turn, contributed to children’s invented spelling skills. Another explanation is that the improvement in the literacy tasks would be due to the EF resources required in performing the tasks (see also Shaul and Schwartz, 2014). The writing task requires a number of highly synchronized skills such as phonemic awareness, grapheme-phoneme correspondence, visual perception, and grapho-motor skills. For example, learning to write words requires holding the representations of letter-sound correspondence in mind, and at the same time retrieving the shape of the letters while writing; furthermore, children must inhibit one letter over the other, such as when learning the letters “c” and “k” in English or phonetically similar letters such as “d” and “t” in Italian. The synchronization of these multiple skills demands a great involvement of EF.

It may be also possible that the increase in EF, in the trained group, allowed the children to benefit more from the educational activities, by improving their cognitive control and consequently making them more ready to learn. For example, early EF were found to support active and positive involvement in classroom tasks and self-regulated use of learning strategies and to limit inappropriate behaviors (such as off-task and disruptive behaviors) that interfere with adaptive engagement (Nesbitt et al., 2015; Nelson et al., 2017).

Concerning math pre academic skills, the interaction between Group (experimental and control) and Time (pre- and post-test phases) was not significant. It is possible that a ceiling effect in one of the two math tasks (Digit Comparison) may have prevented to detect an improvement. Nevertheless, the mediation analysis revealed a significant indirect effect. The interference suppression composite score, enhanced by the training, accounted for math composite score. According to Hayes (2009) given that the total effect is the sum of many different paths of influence, it is possible to detect a significant indirect effect in absence of the total effect. Indeed, we need to be cautious in assuming that the improvement observed in one pre academic skill domain (literacy vs. math) may be due to the specificity of the domain. Several studies’ results support the idea of a domain general association between EF and pre academic skills (e.g., Allan and Lonigan, 2011; Fuhs et al., 2015). Concerning the role of inhibition on math achievement, several studies suggested that inhibition accounts for both pre-academic skills (i.e., Lan et al., 2011; Purpura et al., 2017) and for complex math acquisition such as problem solving (i.e., Passolunghi and Siegel, 2001; Khng and Lee, 2009; Viterbori et al., 2017). Nevertheless, previous studies on pre-academic skills focused mainly on response inhibition (i.e., Purpura et al., 2017), whereas our results revealed a significant association between the ability to suppress interference and the acquisition of Arabic numerals.

### Limitations and Future Directions

Finally, the results are promising and indicate that it is possible to foster the development of different aspects of EF with relatively simple interventions. Nevertheless, the current results should be considered in the context of the study limitations. First, in this study we did not evaluate whether the gains in EF evident in the trained group endured over time, or whether they were associated with achievement in Grade 1. Moreover, given that mediator variable (EF skills) was assessed at the same time point as the outcome measure (pre-academic skills), far transfer need to replicated with data measured at distinct time points. Second, although we were interested in verifying if the training by Traverso et al. (2015) enhance children EF more than regular activities that children usually perform, it is not possible to exclude the Rosenthal effect. Moreover, although this type of training, such as the one developed by Tominey and McClelland (2011) aims to target more directly the EF than the long term curricula, such as Tools of the Mind, which are comprehensive in nature, further studies may address which aspects of this type of training accounts for EF improvement. Indeed, although we suppose that the core aspect of the training were the activities that changed every session and required higher level of cognitive control, the training included other elements such as role playing, metacognitive activities and an adult that actively supported children’s self esteem, therefore it could be interesting to understand the relevance of these aspects. Finally, it may be particularly helpful to verify the effect of this type of intervention with children at risk, such as children with low EF due to social disadvantage (Farah et al., 2006). It should be noted that children from disadvantaged socio-economic backgrounds are more likely than their peers to have lower EF, which in turn contributes to lower academic achievement in Grade 1 (Nesbitt et al., 2013). Hence developing interventions suitable for educational services attended by this population of children could reduce disparities at school entry level and reduce the negative effects of poor self-regulation. Compared to other kinds of training, the one we described appears to be particularly suitable for this population because of its play-based approach, its low costs and its ease of administration.

In conclusion, this study confirms the effectiveness of a school-based intervention that addressed EF in 5-year-old children and indicates that teachers with minimal training may significantly foster the development of EF. In addition, the study shows promising results concerning the possibility of cross- domain transfer to pre-academic skills. Given the predictive association between EF and later achievement, interventions that begin in the preschool period may lead to better outcomes by increasing school readiness. The development of low-cost EF training feasible for educational settings should be considered a priority for prevention research.

## Data Availability

The datasets generated for this study are available on request to the corresponding author.

## Ethics Statement

Ethical review and approval was not required for the study on human participants in accordance with the local legislation and institutional requirements. Written informed consent to participate in this study was provided by the participants’ legal guardian/ next of kin.

## Author Contributions

LT, PV, and MU revised the literature on EF development and EF training, conceived and designed the experiment, and read and approved the final manuscript. LT collected the data and wrote the first draft of the manuscript that was revised by PV and then by MU. LT and MU performed the analysis.

## Conflict of Interest Statement

The authors declare that the research was conducted in the absence of any commercial or financial relationships that could be construed as a potential conflict of interest.

## Footnotes

1 The training is available at www.autoregolazione.org.
