# Supplementary material for: Effectiveness of an Executive Function Training in Italian Preschool Educational Services and Far Transfer Effects to Pre-academic Skills
Source: Front Psychol. 2019 Sep 10;10:2053. doi: 10.3389/fpsyg.2019.02053 (PMC6748352; doi:10.3389/fpsyg.2019.02053)
Supplement: Supplementary file 1 [file Presentation_1.pdf]

THE ADVENTURES OF CHUCKY AND NELLY

12 activities to stimulate
self regulation
in children

by
Laura Traverso, Paola Viterbori e Maria Carmen Usai
University of Genoa
Department of Education Sciences
lauratraverso4@gmail.com

FRAMEWORK

Thinking before you act is a fundamental capacity that allows us to insert a useful space in between our impulse, the external stimulation and our action. This space allows us to verify whether our answers are useful in reaching our objectives and whether they are in harmony with the context in which we live.

During the developmental stages, the brain learns many behavioural models, for example walking, that are automatically carried out every day without great attentive resources. Nonetheless, in the case of a new or difficult situation, the brain should be able to interrupt this automatism, and allow us to consciously regulate out behaviour. At the same time, whenever we have to learn something new, we should be able to pay attention, remember the information and regulate our behaviour. These capacities belong under the dominion of self-regulation.

Adults know the importance of this fundamental capacity in our lives and they know how tiring it is when this ability is compromised. Children need to learn to develop this capacity, in order to be able to learn, to be able to handle their acquired competences better, and to reach their goals. The development of self-regulation allows children to manage themselves, by learning to control their impulses, develop their attention capacity and their memory.

Recent research has shown the possibility to stimulate the development of self-regulation in children, by means of playing. The programme "The Adventures of Chucky and Nelly consists in training the ability to self-regulate in children of five-year-old, engaging children in game activities.

What is it about?

There are 12 activities to be performed three times a week for a month, according to their prescribed order. Each activity lasts approximately 30-45 minutes. The teacher can proposes the activities in the normal school context to a small group of five children. In order to stimulate the development of the cognitive capacity the activities are to small groups of five children. Nevertheless, the activity can be adapted to larger group of children.

What are the kinds of activities?

The teacher asks the children to help two elves, Chucky and Nelly, who have been turned into a cat and a mouse because they were distracted during magic class. Before they can turn themselves back into elves, they have to pass ten magic tests. Then Whizzy the Wizard will give them the Super Magic Attention Potion that will turn them back into Chucky and Nelly. The two elves will be very happy because as elves they can train themselves to be great Magicians. The two elves will thanks the children for their incredible help.

What need to perform the activities?....Material

The materials needed for this course are cheap and easy to get a hold of. For example: coloured cardboard, paper, paper tape, felt tipped pens, pieces of material, stuffed toys, a water bottle, paper plates, bottle lids.

What need to perform the activities? ….Skills

The activities require a teacher who has good self-regulation abilities and can thus be a role model for the children. The teacher must be able to ask the children for help in passing the tests, as well as their full participation. He/she mustn't criticise the children, but instead reinforce only their positive behaviour and their teamwork. The teacher is not the chief, she need the help of the children to pass the Magic Test to help Chucky and Nelly.

If a child is not able to manage the cognitive requests of the game and the other children start to criticise him/her, the teacher will reinforce his/her and the teacher will remember that it is necessary a teamwork to pass the test. We have to help each other or we will fails.

If a child doesn't behave, the teacher can remind him/her that the group needs his/her good behaviour and participation to pass the test. The teacher should sympathise with the child, saying that sometimes it is difficult to follow the rules (or whatever the child isn't able to do). If the child continues to misbehave, the teacher can tell the child that he/she will be removed from the game. If this happens, the teacher mustn't remind the child of his previous misbehaviours in the next activity, but instead the child must be allowed to restart with a clean slate.

IN ORDER TO STIMULATE THE DEVELOPMENT OF SELF-REGULATION IN THE CHILDREN….

IN ORDER TO TRAIN CHILDREN SELF REGULATION…

EACH ACTIVITY REQUIRE THE CAPACITY FOR SELF-REGULATED
Each activity comprehends a new and different group game (The Magic Test) that is more complex than the previous one. The games cannot be played automatically or without thinking, they requires cognitive control and self regulation. The activities require the children to actively participate, whether they are players, directors or referees. The children are required to change roles during a single game. In each activity, each child will be a player then the director and then the referee. When children perform the game they train their self regulation ability.

IN ORDER TO HELP CHILDREN IN TRAINING….

EACH AVTITIY HAS A FAMILIAR AND PREDICTABLE STRUCTURE FOR THE CHILDREN
Beginning: each activity begins by dancing to a song titled "The Super Magic Careful Song" and by answering three questions; why are we here? What have we done so far? What are we going to do now?
Game (The Magic test)
Ending: each activity ends by celebrating the fact that the children have passed the test and that the children did their best. This is done by colouring in the self-assessment chart and by reflecting on the strategies that the children used to pass the test.

THE ACTIVITY ARE PERFORMED WITHING A NARRATIVE OUTLINE
In order to keep the children motivated, each activity takes place within a fantasy style story of which the children themselves become characters.

THE ACTIVITIES STIMULATE GROUPS WORK
The tests are passed by the group, not the individual. If a child has self regulation difficulties, the group must help him/her.

THE ACTIVIY ARE PERFORMED BY USING CONCRETE OBJECTS
The use of materials help the children in regulating their behaviour.

EACH ACTIVITY ENDS WITH A REFLECTION ON METACOGNITION AND SELF REGULATION STRATEGIES
Each activity includes a moment to reflect on the abilities that were needed for each child to pass the test.

IN ORDER TO SUPPORT CHILDREN IN THINKING THAT THEY CAN SELF REGULATE….

POSITIVELY REINFORCE THE CHILDREN'S SELF-IMAGE
At the end of each activity there is a moment to celebrate that each child passed the test and that they all did their best. The activities are limited (to about 30 minutes) so that the children to not become tired out, but instead are able to feel competent in their capacity for self-control.

PROPOUSE CHILDREN COMPLEX GAMES THAT REQUIRE THEIR SELF REGULATION ABILITY AND THEIR ACTIVIE PARTECIPATION

SUPPORT CHILDREN IN THE TRAINING WITH

- ✓ ROUTINES
- ✓ NARRATIVE STORY THAT PROVIDES MOTIVATIONAL AND EMOTIONAL INVOLVEMENT,
- ✓ CONCRETE MATERIALS

HELP CHILDREN IN UNDERSTANDING HOW TO SELF-REGULATE, DISCUSS USEFUL SELF REGULATION STRATEGIES

LET CHIDLREN DO EXPERIENCE AND SUPPORT CHILDREN WITH POSITIVE REINFORCEMENT

START
CHUCKY, NELLY AND THE MAGIC CAREFUL SONG

MAGIC TESTS
1. CHUCKY, NELLY AND THE MAGIC FROG
2. CHUCKY, NELLY AND THE MAGIC POTION
3. CHUCKY, NELLY AND THE MAGIC MIRROR
4. CHUCKY, NELLY AND THE FORGETFUL ELEPHANT
5. CHUCKY, NELLY AND WHIZZY THE WIZARD'S HOUSE
6. CHUCKY, NELLY LOOKING FOR WHIZZY THE WIZARD
7. WHIZZY THE WIZARD WANTS TO KNOW IF CHUCKY AND NELLY CAN CONTROL THEIR ACTIONS
8. WHIZZY THE WIZARD WANTS TO KNOW IF CHUCKY AND NELLY CAN PAY ATTENTION
9. WHIZZY THE WIZARD WANTS TO KNOW IF CHUCKY AND NELLY CAN REMEMBER DETAILS
10. WHIZZY THE WIZARDS WANTS TO KNOW IF CHUCKY AND NELLY CAN THINK BEFORE THEY ACT

THE END
FINALLY CHUCKY AND NELLY ARE ELVES AGAIN, THANKS TO THE CHILDREN'S HELP!

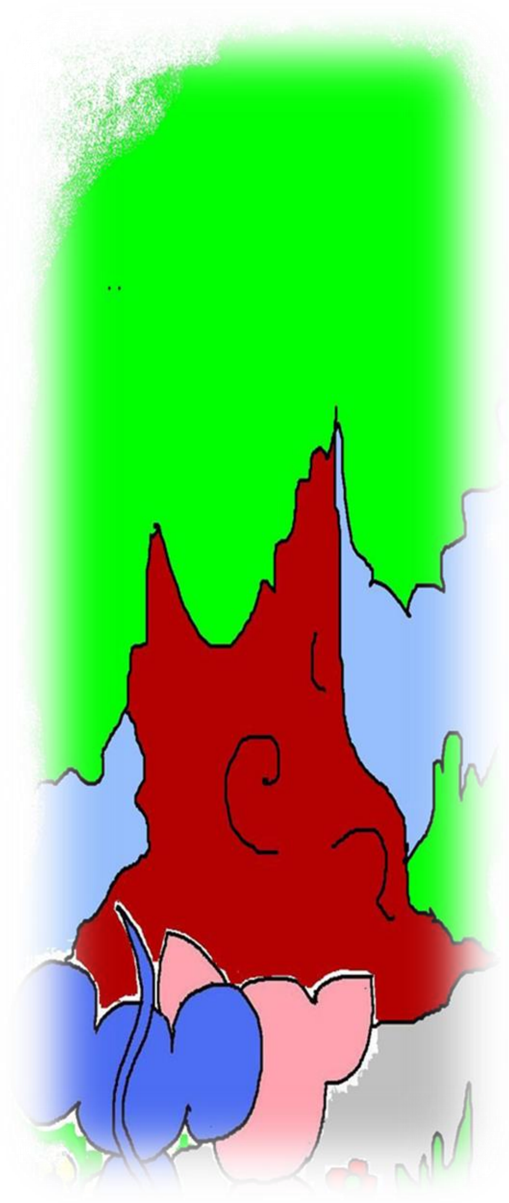

GOOD JOB!

START
Chucky, Nelly
and the Magic Careful Song!

MATERIALS

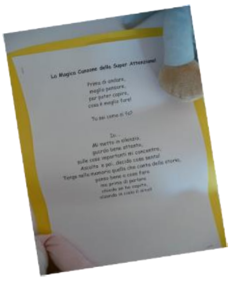

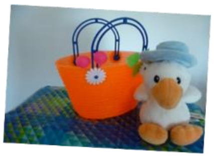

- ✓ A stuffed bird in a basket, hidden away at first.
- ✓ Six place cards (post-its, cushions, etc…), one for each child and one for the teacher, placed in a circle on the floor.
- ✓ A print out of slide 1, glued on a piece of coloured cardboard, rolled up, tied with a string and hidden in the basket with the stuffed bird.
- ✓ A stuffed cat
- ✓ A stuffed mouse
- ✓ Print outs of slide 2 (self-assessment chart), one for each student.
- ✓ Felt tipped pens.
- ✓ A box that can be used to put the activity materials in.

THE GAME

Listen to the Chucky and Nelly's story and learn the words and the movements of the Super Careful Song.

THE GAME'S INSTRUCTION

Hello. Come sit in a circle. Can you see your place card? Good!
I called you here today because I have to ask you a big favour…
I have two small friends who are in a lot of trouble. Would you like to hear why?
Chucky and Nana are two friendly little elves, about this tall (show how tall they are). They are small but they look just like children. They are five years old and they go to pre-school, just like you!
But their school is called the Magic Rainbow Tree in the Enchanted Forest. At this school, they learn lots of magic things that will help them go to school the following year.
But Chucky and Nelly aren't very good at paying attention. They know they have to listen to the Fairy Teacher, but they can't help but talk and play during the lesson. Every now and then they fight and make a big ruckus. Because of this they don't listen to the Fairy Teacher and make a mess of things.
Yesterday they were making a magic potion
Instead of following the rules they got the notion
To fight and make a big commotion
That nearly a very loud explosion
Boom!
By accident, Chucky and Nelly were transformed into… a mouse and a cat! Oh no!
They were very sad because mice and cats can't play together. They want to go to school, but they can't go unless they're elf children. They promised the Fairy Teacher that they would be more careful next time. The Fairy Teacher said that was a good idea.
When you get distracted it's easy to make a big mess.
The Fairy Teacher said that there is a magic way to learn to pay more attention. But it's a big secret! Come close and I will whisper it to you (whisper)
You need to pass ten magic tests. If Chucky and Nelly are able to pass these tests, they can go see Whizzy the Wizard, who will give them the magic potion that will turn them back into elves.
But the tests are very difficult!
Do you want to help them pass the tests so that they can go see Whizzy the Wizard so that they can get the potion to turn themselves back into elves?
Yes? Good!

The Fairy Teacher will be so happy that you have agreed to help that she will surely have sent her helper who can use magic to help us to learn to pay attention.
Do you know who her helper is?
It's Harry the Hummingbird!
But if you want to meet him we have to stay very quiet and still, otherwise he will be scared ad fly away.
Here he is (get the stuffed bird)!
Harry the Hummingbird told me that he has come to teach us the Magic Careful Song. It's a magic song that will help us to pass the tests. But in order to learn the song we first have to put our voices in this basket (pass the basket around and make the action of putting your voice in the basket. Show the children that you move your mouth without making any noise). You can speak only if you are holding Harry the Hummingbird.
Whoever wants to learn the Magic Careful Song must tell Harry the Hummingbird. But only when you are holding him. I want to learn the Magic Careful Song. And you? (pass the bird on to the children)
Good! Before we leave on our journey we must learn the Magic Careful Song. We need to stand on this magic piece of paper (the rolled up cardboard). Try to copy me (show the children the actions for the song, first slowly, then a little bit faster).

WORDS	ACTIONS
The Super Magic Careful Song	Wave your hands in front of your face then make a big circle with your arms.
Be careful what you do	Hop forward on one leg, then balance there
You must first think it through!	Point to your temple, still balancing.
How do we do it?	Stand back on two legs.
We…	Put your hands out, palms up, as a question
Stop! Look!	Put your hand out in front of you, then shade your eyes with your hand.
Listen! Think!	Cup your ear to hear better. Point to your temple.
Stop, don't make a sound	Put your hand out in front of you, put your finger to your lips
Look all around	Shade your eyes again, pretending to look around
Listen, stay on guard	Cup your ear to hear better. Then make a salute.
Think very hard!	Point to your temple, then cringe your face in concentration.
Stop! Look!	Put your hand out in front of you, then shade your eyes with your hand.
Listen! Think!	Cup your ear to hear better. Point to your temple.
Now that you've thought it through	Give a thumbs up
Decide what you want to do!	Put your hands to your heart.
Remember what you know	Point to both temples
So that you know which way to go.	Point with your outstretched arm all around the room
But before you speak you should	Put your finger to your lips
Make sure you've understood.	Point up, in an ah-ha way.
Then, don't be shy Smile Stick your hand up high!	Put your hand up in the air.

Well done!
The Teacher give to each child the slide 2 and ask them to color with different colors according if they feel that they have been able to pay attention (yellow), or it was a little difficult (blu) or very hard (red). While they do with the colors that they want, the teacher talk about what the song means: why does the song say… ? Do you think this is useful…? Give some examples: when there are lots of us and one of our friends is talking, should we decide to listen on to them?
Great Job!!! Now we are ready to help Chucky and Nelly!!!! Thanks!!!!

THANKS TO THIS ACTIVITY THE CHILDREN…

Because of this activity, the children… talk about useful strategies to increase their capacity of self-control and therefore become able to filter information that comes from outside, inhibit impulses, remember important information and elaborate, summarise and think before they act!

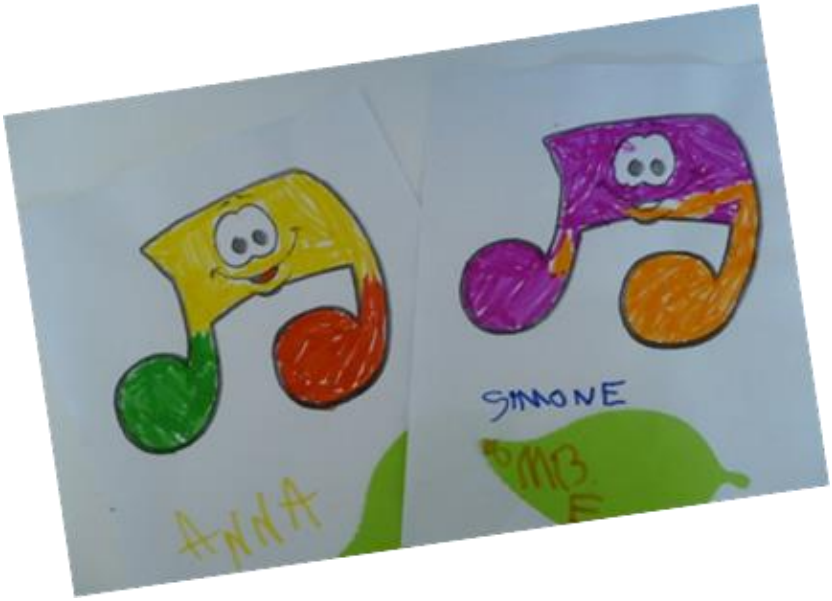

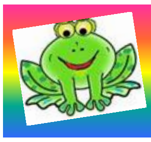

First MAGIC TEST
Chucky, Nelly and the Magic Frog!

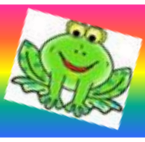

MATERIALS

- ✓ Printouts of slides 1-14 (cut 11-12-13).
- ✓ Two pieces of coloured cardboard (different colours) to stick on the printouts for the Elf Director and the Elf Referee.
- ✓ A magic wand (made from a pen, pencil or other stick)
- ✓ Two pieces of different coloured material for the Director and the Referee elves to use as capes. Two paper hats, preferable the same colour as the pieces of carboard.
- ✓ Printouts of the self-assessment table (slide 14, 1 for each student).
- ✓ Felt tipped pens to colour the self-assessment table.
- ✓ A stuffed frog (optional)!

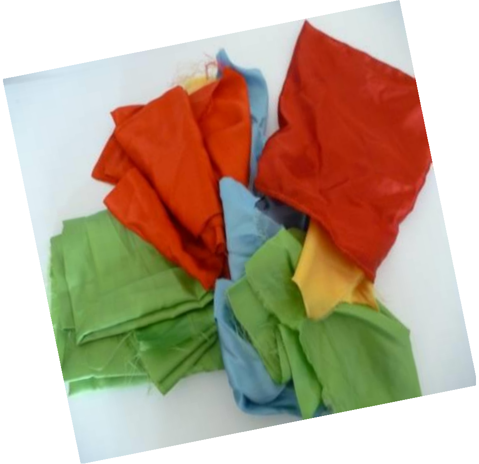

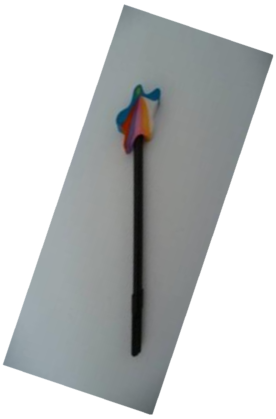

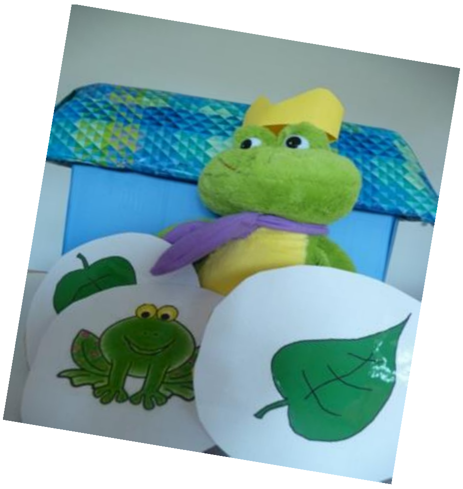

STRUCTURE OF THE ACTIVITY

Introduction

Hello everybody!
Come sit in a circle! (use place cards for each student until they are able to find their place on their own).
Do you remember why we're here? …. (help the kids answer) Yes! We're here to help Chucky and Nelly.
What have we done so far? … Yes! We met Chucky and Nelly!
Do you know what we're going to do now? … We have to pass the first magic test!
But first: The Magical Careful Song!

Game

Do you know what the first magic test is? Today … (explain the game on the following paragraphs).

Ending

We passed the first test! Now we're all going to take this picture (slide 14) and colour it in depending on how well you think you paid attention. Colour in the the smiling frog if you think you paid attention very well. Colour in the straight-faced frog if you think you paid attention, but it was a bit difficult. And colour in the sad frog if you don't think you paid much attention. While we're colouring, how do you think we passed the test? (repeat what the children say, sharing any useful strategies e.g.: we kept quiet while we were listening, we whispered to ourselves whatever

we had to remember…). Have you finished colouring? Good! Everyone stand up, one hand above the other. Now say "Magic Frog, we passed the magic test!" Yay!

THE GAME

In this game, the Players have to move according to specific rules when they listen to what the Director says. The Referee has to control if the players perform correctly and he/she has to sign the score. The children are required to change roles.

THE GAME'S INSTRUCTION

Today we have to help Chucky and Nelly with the Magic Frog!
Who is the magic frog?
(say the rhyme together)
The Magic Frog hopped here and there,
But didn't take much care,
Looking for a sunny lily pad.
But when he hopped he didn't look,
And so fell in the icy brook,
Now the Magic Frog is cold and sad!
The game works like this: everyone stand up.
Every time I say "frog", jump (show the kids the action)
And every time I say "lily pad", put your hands on the ground (kneel down).
Let's try: Frog, lily pad, frog, lily pad.
Well done! Now let's see if you're attentive Magicians!
I will say a few words, but you must sit down only if I say "lily pad" and you must stand up only if I say "frog".
Let's practise! Tree, fairy, frog, bee, leaf, lily pad, butterfly. Well done!
Let's practise again! Flower, mushroom, frog, lake, lily pad. Well done!
Let's practise again! Bee, log, tree, frog, ladybug, lily pad. Well done!
Are you ready for the real game? We need two chairs. The Elf Director must sit in this chair, wear the Director's cape and hold up these drawings (slide 1 – 10). The Elf Referee must sit in this chair, wear this cape and hold the magic wand and the score board (slide 14).
The three Elf players must stand in a line in front of the Elf Director and the Elf Referee. Each player must hold a piece of cardboard with either a frog or a lily pad (slide 11-12-13 cut out).
The Elf Director must say out loud which picture he can see in the first row, from here (left) to here (right).
The Elf Referee, together with the Director, has to check to see that:
1. The Elf Player with the frog jumps every time the Elf Director says "frog" and stays dead still when the Elf Director says anything else.
2. And that the Elf Players with the leaves touch the ground every time the Elf Director says "lily pad" and they stay dead still when Elf Director says anything else.
Elf Players, you must look carefully which picture you have, remember it, and hold it so that the Elf Director and the Elf Referee can see them.
If the Elf Players get a whole row right, the Elf Referee must say "Magic Frog" and tick the frog (slide 14). In order to make a tick, every player has to get it right.
We pass the test if we get at least 8 points.
The Elf Director can now start to say out loud the pictures he sees!
After the first page, the Elf Players must swap cards (cut out slides). After two pages, they must rotate the roles (show the kids in which direction to rotate) so that someone else becomes the Elf Director and someone else becomes the Elf Referee.
Do you all understand? Let's begin! (Encourage the kids to manage the game themselves.)
Well done! Help me!
(Once the test has been passed, everyone stands in a circle and holds one hand above the other saying "Magic Frog". Then they colour in the self-assessment chart and reflect on the best strategies to pass the test.

THANKS TO THIS ACTIVITY THE CHILDREN….

They practise their self-regulation, that is their cognitive capacity that helps them focus on useful stimuli and regulate their impulses, in addition to training their memory and focus.

Thinking about the game…

The Elf Players must be able to listen to the director carefully, move only when they hear a certain stimulus, regulate their impulse to automatically react, remember which movement they must do according to the rule and remember what they have (the frog or the lily pad picture)

The Elf Director and the Elf Referee must organise and monitor the activity. The Elf Director must name the pictures by quickly recalling the correct vocabulary, correctly follow orders and check the Elf Players' answers.

The Elf Referee must check the progress of the test, make sure that the Elf Players answer correctly, remember the right answer and award points accordingly.

The constant exchange of roles stimulates the children's flexibility and pushes them to pay close attention.

The narrative outline gives motivation and the celebration at the end of the game reinforces a positive self image for the children.

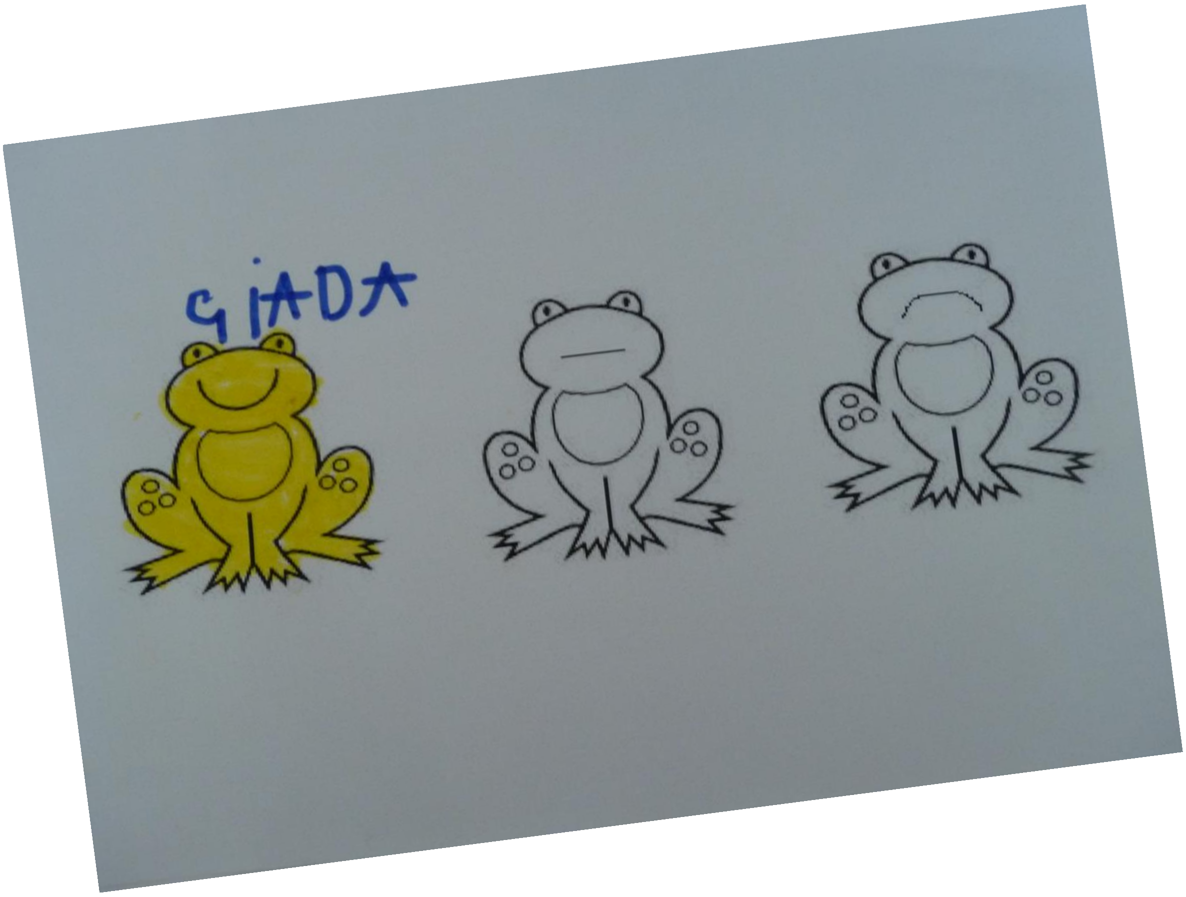

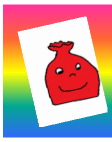

2nd MAGIC TEST
Chucky, Nelly and the Magic Potion!

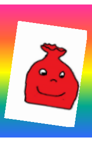

MATERIALS

- ✓ Printouts of slides 1-14 (cut out the 'ingredient' cards on slide 11-12-13).
- ✓ A trey to put the cut outs in.
- ✓ Paper tape to make three parallel lines of about 1,50m and a perpendicular starting line.

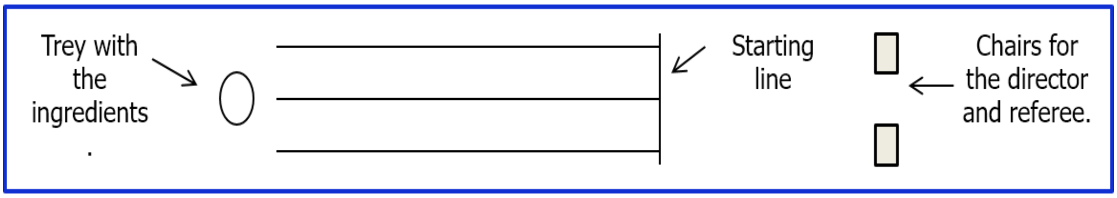

- ✓ A magic wand (made from a pen, pencil or other stick)
- ✓ Two pieces of differently coloured cardboard that the Elf Director and Referee can use to hold up the slide printouts (slide 1-10 for the Director and 14 for the Referee).
- ✓ Two pieces of different coloured material for the Director and the Referee elves to use as capes. Three pieces of material (one yellow, one red and one green) and another piece of material that's the same length as the starting line.
- ✓ Printouts of the self-assessment table (slide 15, 1 for each student).
- ✓ A bell

STRUCTURE OF THE ACTIVITY

Introduction

Welcome back!
Come sit in a circle! (use place cards for each student until they are able to find their place on their own).
Do you remember why we're here? …. (help the kids answer) Yes! We're here to help Chucky and Nelly.
What have we done so far? … Yes! We helped Chucky and Nelly!
Do you know what we're going to do now? … We have to pass the second magic test!
But first: The Magical Careful Song!

Game

Do you know what the second magic test is? Today … (explain the game on the following paragraphs).

End

We passed the first test! Now we're all going to take this picture (slide 21) and colour it in depending on how well you think you paid attention. Colour in the the smiling potion if you think you paid attention very well. Colour in the straight-faced potion if you think you paid attention, but it was a bit difficult. And colour in the sad potion if you don't think you paid much attention.
While we're colouring, how do you think we passed the test? (repeat what the children say, sharing any useful strategies e.g.: we kept quiet while we were listening, we whispered to ourselves whatever we had to remember…).
Have you finished colouring? Good! Everyone stand up, one hand above the other. Now say "Magic Potion, we passed the magic test!" Yay!

THE GAME

The Players have to control their movement and focus on specific information (the ingredients) and be able to remember it. The Director shows the ingredients that must be remembered. The Referee controls if the Players perform correctly and he/she signs the scores.

THE GAME'S INSTRUCTION

Try to follow me while I walk along this line (saying the rhyme)
Slowly we walk along the line,
Like little ants along a vine
If we hear a noise we stop all motion
Then move on slowly on to get the potion
Today we have to help Chucky and Nelly to remember the ingredients for the potion.
The Elf Director, wearing his cape, must hold this piece of cardboard with these pieces of paper (slides1-10).
The Elf Referee, wearing his cape, must hole this piece of paper, with the score card (slide 14) and the Magic Wand.
Then the Elf Players must get on their marks behind the starting line.
The Elf Players, each with a different colour piece of material, must first check the ingredients on the Elf Director's pieces of paper.
The Elf player with the yellow piece of material must remember the yellow ingredients. The player with the green piece of material must remember the green ingredients and the player with the red piece of material must remember the red ingredients.
Once you have memorised the ingredients, you must go fetch the ingredients by walking along this line. But make sure you walk at the same pace, holding this piece of material between all of you. If one of you get to the trey before the others, he cannot take the ingredients.
When you get to the trey, grab your ingredients and come back the same way.
Be careful! If the Elf Director rings the bell, you have to stand still like a statue.
The Elf Director and the Elf Referee will check to see that you have taken all the correct ingredients. If you do it right, the Elf Referee can put a tick on the score card and say Magic Potion!
After two rounds the roles are rotated clockwise. After the first round, the Elf Players must remember their colour and keep their piece of material in their pocket.

THANKS TO THIS ACTIVITY THE CHILDREN…

They continue to work on controlling their impulses and filtering useful information, training their memory most of all.
Thinking back on the game…
Walking along the line, the children have to go slowly, balancing carefully, and waiting for the others. This continuous regulation of movement, including the rule to stop if the Director Elf rings the bell, exercises their capacity for self-regulation and impulse control.
At the same time, the Elf Players must be able to focus their attention only on the ingredients with their colour, remember them while walking along the line so that they can bring them back to the Elf Director.
The Elf Director and the Elf Referee must organise and monitor the game.
The Elf Director has to check the Elf Players while they memorise their ingredients. He also has to remember to ring the bell while the Players are walking along the line.
The Elf Referee has to monitor the progress of the game, check to see if the Elf Players brought back the correct ingredients, remember how many got it right and which cards, and appropriately award points.
The constant exchange of roles stimulates the children's flexibility and pushes them to pay close attention. The narrative outline gives motivation and the celebration at the end of the game reinforces a positive self image for the children.

3TH MAGIC TEST
Chucky, Nelly
and the Magic Mirror!

MATERIALS

- ✓ Paper tape to draw a castle on the ground. Draw a door, five rooms for the Elf Players and a roof (a triangle) for the Elf with the Magic Mirror.

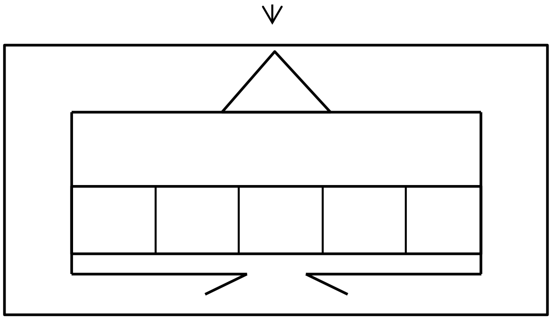

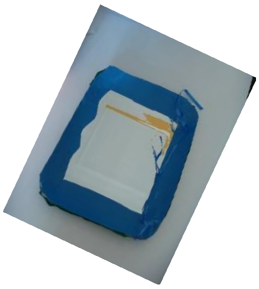

- ✓ Self-assessment chart (slide 1, 1 copy per student) and felt tipped pens.

STRUCTURE OF THE ACTIVITY

Introduction

Hello everyone!

Come sit in a circle! (use place cards for each student until they are able to find their place on their own).

Do you remember why we're here? …. (help the kids answer) Yes! We're here to help Chucky and Nelly.

What have we done so far? … Yes! We helped Chucky and Nelly!

Do you know what we're going to do now? … We have to pass the third magic test!

But first: The Magical Careful Song!

Game

Do you know what the third magic test is? Today … (explain the game on the following paragraphs).

Ending

We did very well at passing the third magic test! Now we're all going to take this picture (slide 1) and colour it in depending on how well you think you paid attention. Colour in the smiling mirror if you think you paid attention very well. Colour in the straight-faced mirror if you think you paid attention, but it was a bit difficult. And colour in the sad mirror if you don't think you paid much attention. While we're colouring, how do you think we passed the test? (repeat what the children say, sharing any useful strategies).

Have you finished colouring? Good! Everyone stand up, one hand above the other. Now say "Magic Mirror, we passed the magic test!" Yay!

THE GAME

The Players have to perform the movements that the Director with the Magic Mirror shows according to the game's rules. The Referee controls and sign the scores.

THE GAME'S INSTRUCTION

Come inside… (while the children are going inside the castle, say this rhyme)

The Fairy Castle in the woods
Is overflowing with magic goods
Hurrah! We've found the Magic Mirror
But as we look into its shimmer
The Mirror says "You must now do
All that I tell you to
But if I say the Magic Word
You must do the reverse"
Let's stand up, whoever is holding the Magic Mirror (first the teacher), must stand here (the triangle) while each of the Elf Players must stand in their rooms, facing the Magic Mirror.
Remember what the rhyme says! You must listen to the Magic Mirror and do what it says.
Let's see if you can! (Make the movements and see if the children can copy you.)
The Magic Mirror says: hands up, touch the ground, put your arm out (to the right), put your other arm out (to the left).
Well done! Now be careful. Because if I say the Magic word "*Babush*" before I say an action, you have to do the *opposite* of what I do (show the children the movements above and help them to understand what the opposite of each movement is). For example, if I say "*Babush* hands up!" you have to touch the ground. If I say "*Babush* hands down!" you have to put your hands up! If I say "*Babush*, put your right hand out!" you have to put your left hand out. If I say "*Babush*, put your left hand out!" you have to put your right hand out.
Let's try! (Do some actions, saying "*Babush*" before two of them.)
Well done!
Now take turns to be the Magic Mirror, you have to say and show three movements. You must say "*Babush*" before one of these movements.
Once the Magic Mirror has finished his three actions, he must say if the Elf Players did the actions correctly, and then pass the mirror on to the Elf Player in the first room. Everyone moves up a room, and the Elf Player who was first holding the mirror goes to the last room. Let's begin! (Help the children to play the game.)
Well done!
We did everything the Magic Mirror said.
(The teacher who played with the children takes the mirror back and puts it in the triangle.)
Now I'm going to show you some more actions and when I've finished I'm going to touch the Magic Mirror. You have to copy my actions in the same order. You have to remember which actions I did and copy them. You can't move until I touch the Magic Mirror again. (If the children are tired they can sit down.) Are you ready?
(say and show:) 2 actions (e.g.: touch your head, clap your hands); 2 actions; 3 actions; 3 actions, 4 actions.
Now take turns to come in my place. You have to do two actions, then three. You have to check to see that the Elf Players do them correctly.. (The children take turns again, rotating until the Mirror is back to the beginning, with the teacher.)
Well done! Now for the last and most difficult part!
Now I'm going to do some actions again, but if I say "Babush" you have to do the opposite.
So if I say "Babush" and then I touch my head and then I clap my hands you have to *first* clap your hands and *then* touch your head. Got it?
I'm going to do two actions four times and I'll say "Babush" twice.
I'm going to do three actions four times and I'll say "Babush" twice.
Well done! We have passed the Third Magic Test!
As usual give the teacher gives to each child the self assessment chart and discuss self regulation strategies.
Everyone stand in a circle, put one hand on top of the other and say "Magic Mirror! Yay!"

THANKS TO THIS ACTIVITY THE CHILDREN…
They continue to work on controlling their impulses, and especially train their short term memory (which we need in order to remember something said in the moment) and their working memory

(which we need in order to elaborate information). The children begin to exercise their cognitive flexibility, as well as their cognitive capacity that helps them to find new alternatives instead of persisting with the same behaviour.

Thinking back on the game…

The Elf Players have to remember the actions and repeat them. When they hear the magic word, they have to be able to suppress the impulse to copy the Elf Player with the Magic Mirror and do the opposite instead. In addition when they have to do more opposite actions they have to keep in mine all the actions and do the opposite at the same, thereby exercising their working memory.

When the children are in charge of the Magic Mirror, they have to be able to imagine a series of actions. This exercises their cognitive flexibility as they cannot simply repeat the same action over and over. It also helps them focus their attention on the others.

The constant exchange of roles stimulates the children's flexibility and pushes them to pay close attention. The narrative outline gives motivation and the celebration at the end of the game reinforces a positive self image for the children.

4TH MAGIC TEST
Chucky, Nelly and the Forgetful Elephant!

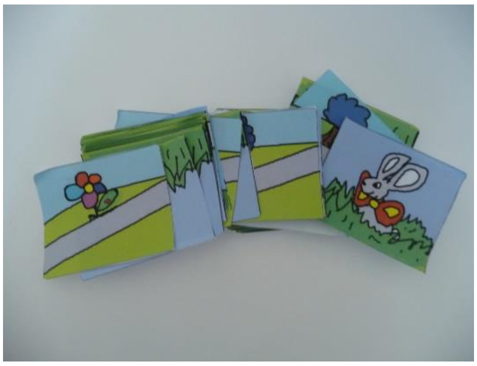

MATERIALS

- ✓ Printouts of slides 1-5, with the images cut out (one for each child)
- ✓ Printouts of slides 5-6-7 (one per participant)
- ✓ Printout of the self-assessment chart (slide 8, one per participant).
- ✓ Felt tipped pens for the self-assessment chart.
- ✓ A stuffed elephant if you want.

STRUCTURE OF THE ACTIVITY

Beginning

Hello everyone!
Come sit in a circle! (use place cards for each student until they are able to find their place on their own).
Do you remember why we're here? …. (help the kids answer) Yes! We're here to help Chucky and Nelly.
What have we done so far? … Yes! We passed the Magic Mirror test.
Do you know what we're going to do now? We have to pass the fourth magic test!
But first: The Super Magic Attention Song! (sing and dance together)

Game

Do you know what the fourth magic test is? Today … (explain the game on the following paragraphs).

Ending

You've been so good, we've passed the fourth magic test! Now we're each going to take this drawing (slide 8) and choose three colours. (Once the children have chosen their three colours, tell them which colour corresponds to which state of attention.) While we're colouring in, tell me if you think we've past the test? (repeat what the children say, sharing any useful strategies e.g.: we kept quiet while we were listening, we whispered to ourselves whatever we had to remember…).
Have you finished colouring? Good! Everyone stand up, one hand above the other. Now say "Never Again Forgetful Elephant, we passed the magic test!" Yay!

THE GAME

The children have to listen to a story and then they have to find out which is the correct picture that was described in the story. Then a Child has to propose a new story and the others have to manage the information they have listen to.

THE GAME'S INSTRUCTION

Today Chucky and Nelly have to help the Forgetful Elephant remember the way home.
Let's start the test!
Now I will read what the Forgetful Elephant remembers seeing and you have to find it out of the pictures that I will give you.
Before we begin reading, take one of these pages (slide 5). You will have to place the pictures that I will give you on this page.
Do you understand? Remember what the Forgetful Elephant says (make a funny elephant voice while you say the rhyme).
After going out my door

I followed a road straight as pin
And saw a blue tree along side it.
Here are the pictures (hand out the cut outs of slide 1) Which one is correct?
Do you remember what the Elephant said? (Let the children remember on their own, then repeat the rhyme). Well done! Now put that one aside. Give me the left over pictures.
Now listen:
Then I passed a colourful flower
It had a leaf, small and green
And a ladybug could be seen
Here are the pictures (hand out the cut outs of slide 2) Which one is correct?
Do you remember what the Elephant said? (Let the children remember on their own, then repeat the rhyme). Well done! Now put that one aside. Give me the left over pictures.
Now listen:
Walking through a beautiful meadow
I saw a grey rabbit stop to think
He had a red and yellow bow
His ears were very long and pink
Here are the pictures (hand out the cut outs of slide 3) Which one is correct?
Do you remember what the Elephant said? (Let the children remember on their own, then repeat the rhyme). Well done! Now put that one aside. Give me the left over pictures.
Now listen:
On my way to the Enchanted Wood
A little mushroom before me stood
His stem was as yellow as the sun
His bright pink cap has just one
dot of such a deep hugh
But it wasn't black, it was blue!
Here are the pictures (hand out the cut outs of slide 4) Which one is correct?
Do you remember what the Elephant said? (Let the children remember on their own, then repeat the rhyme). Well done! Now put that one aside. Give me the left over pictures and we'll check.
Take all the leftover pictures. For the last time I will read out the Forgetful Elephant's last story and you have to tell me if you have chosen the right pictures. Then you can put them on your page. (slide 5)
Well done! Now everyone gets a chance to be the Forgetful Elephant. You can give me back your pages. (slide 5)
The first Elf Players can come sit here in my seat and must make the Elephant voice while you say another two things you've seen, choosing from the four pictures.
We have to tell the Forgetful Elephant how to get back!
If the Elephant says he first say the rabbit and then the flower, we must tell him that to go back home he must first look for the flower and *then* the rabbit! Let's begin! Here are the pages you can use (give each participant a copy of slide 6 and take back slide 5)(let everyone have a turn to be the Forgetful Elephant)
Well done! Now let's try with three pictures! (give each participant a copy of slide 7 and take back slide 6)(let everyone have a turn to be the Forgetful Elephant)
Well done! We've managed to make the Forgetful Elephant a master of memory! Now he will never get lost again! Well done! We've passed the test!

THANKS TO THE ACTIVITY THE CHILDREN….

They practise paying attention to information, concentrating on relevant data, keeping them in mind and developing them, that is they are using their working memory. In addition, they continue to train their inhibitory capacity and their cognitive flexibility.
If we think back on the game…
When the children listen to the Forgetful Elephant they have to keep the given information in mind so that they can concentrate on the useful information to find the correct card.

To find the correct card they have to compare the different characteristics of the drawings, continuing to train their memory. Furthermore, to pass the test they have to be patient, listening to the end, repressing the impulse the impulse to take a card before having received all the necessary information.
When the children area acting the Forgetful Elephant, they have to be able to control the tone in their voice and at the same time suggest to the other children different routes taken by the elephant, exercising their cognitive flexibility.
As already stated, the flexibility of the children and is also simulated by the constant change of roles.
Lastly, the narrative outline gives motivation and the celebration at the end of the game reinforces a positive self image for the children.
Passing the test always depends on the group, never on the individual. In this way the individuals are on one hand upheld, and on the other hand each feels the responsibility of passing the test, so they feel it is important to encourage the others to involve themselves.

5TH MAGIC TEST
Chucky, Nelly
and the Whizzy the Wizard's house

MATERIALS

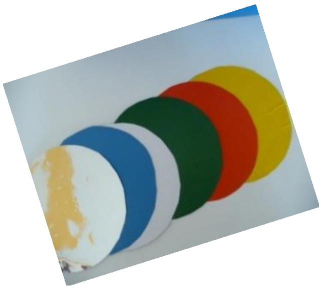

- ✓ Paper tape to make a 5 X 5 chess board on the floor. (each square should be big enough for a child to stand in)
- ✓ Five sets of felt tipped pens , yellow, blue, green and red
- ✓ Coloured cardboard (yellow, blue, red, green, which and silver)
- ✓ A printout of the slide 3-4-5 one for each participant)

STRUCTURE OF THE ACTIVIY

Beginning

Hello everyone!

Come sit in a circle! (use place cards for each student until they are able to find their place on their own).

Do you remember why we're here? …. (help the kids answer) Yes! We're here to help Chucky and Nelly.

What have we done so far? … Yes! We helped the Forgetful Elephant!

Do you know what we're going to do now? We have to pass the fifth magic test!

But first: The Super Magic Attention Song! (sing and dance together)

Game

Do you know what the fifth magic test is? Today … (explain the game on the following paraghaps).

Ending

You've been so good, we've passed the fifth magic test! Now we're each going to take this drawing (slide 11) and colour it in depending on how well you think you paid attention. Colour in the the smiling mushroom if you think you paid attention very well. Colour in the straight-faced mushroom if you think you paid attention, but it was a bit difficult. And colour in the sad mushroom if you don't think you paid much attention. While we're colouring, how do you think we passed the test? (repeat what the children say, sharing any useful strategies).

Well done! Everyone stand up, one hand above the other. Now say "Whizzy the Wizzard, we passed the magic test!" Yay!

THE GAME

The game consists of two activities. First the children have to control their movement according to what they listen in order to acquire the felt tipped pens. Secondly, they will have to listen to the Whizzy the Wizzard's house description. They have to remember the details and then they have to colour the picture of the house that was covered when they were listening.

TE GAME'S INSTRUCTION

Now we have to help Chucky and Nelly with another test.

Look at this (give each child a printout of slide 1) Can you see how far we've come with Chucky and Nelly? Remember … (remember with the children the tests you have passed so far, and look ahead on the tests to come). Now Chucky and Nelly have finally arrives at Whizzy the Wizards house!

Do you remember who Whizzy the Wizzard is? That's right! He is the Wizard who Chucky and Nelly were looking for to get the Super Magic Careful Potion to turn them back into children! Now you can put this page away (slide 1), you can colour it in at home, so that we can start today's test.

Whizzy the Wizard will open his door only once we've coloured in his drawing!

Whizzy the Wizard has drawn his house (show the children slide 2). But before we can colour it in we have to wind the colours! Quickly, Elf Players, get into a square on the chess board (show the children the chess board on the floor).
The game works like this: In order to get the drawing and the colours you have to get to here (show the end of the chess board) twice: once to get the drawing of Whizzy the Wizard's house, and a second time for the colours.
Now let's try to get the drawing! (leave a copy of slide 2 for each child at the end of the chess board). Everybody to the start of the chess board!
Now I'm going to say some colours and I'll show you these colourful cards (show them the cards). If you hear and see the same colour, you can jump forward. But if I show you one colour but say another you have to stay where you are. For example, if I say red, but I show you the yellow card, you need to stay still. If I say or show you the colour silver, you must stand on one leg until I say the next colour. Do you understand? If you make a mistake you must take a step back. In order to win this round everyone has to get to the end of the chess board! Let's begin!
(Say out eight card/colour combinations: say the same colour as the card five times, say a different colour twice and say the colour silver once. (For example: white + white card, yellow + yellow card, silver + silver card, red + yellow card, green + green card, blue + blue card, white + white card, yellow + yellow card, green + red card.)
(Repeat this process until all the children have reached the other side of the chess board.) Well done! Now we need to go back to the beginning and do it again to get the coloured markers! I'm going to give each of you a coloured piece of material. You can have the red one, you have have the yellow one, you can have the green one, you can have the blue one, and you can have the white one. When you hear your colour you have to stay still. When you hear any other colour you can jump forward. Got it? Let's begin!
Call out a few colours, in alternative way and show the colour until all the children have reached the other side of the chess board.
Well done! You've won the coloured markers/felt tipped pens!
Now we can colour in Whizzy the Wizard's house! Let's all sit in a circle and turn our pages face down. Now listen to what Whizzy the Wizard says. You have to listen to his instructions and do exactly what he says! Otherwise he won't let us in!
I live in the Colourful Tree in the Enchanted Woods
In front of my house there is a beautiful green garden
Did you understand? Turn over your drawing and colour them in just like Whizzy the Wizard said. Turn the page back over and listen up for the next instruction.
When I'm in, I look out my blue window
Turn the drawing back over. Do you remember what Whizzy the Wizard said? Good. Let's colour it in! As soon as you've finished, turn the drawing face down again and listen.
My door is yellow, not red, just like the star overhead
Turn the drawing back over. Do you remember what Whizzy the Wizard said? Good. Let's colour it in! As soon as you've finished, turn the drawing face down again and listen.
Among the trees you can see my branches are yellow and blue!
Turn the drawing back over. Do you remember what Whizzy the Wizard said? Good. Let's colour it in! As soon as you've finished, turn the drawing face down again and listen.
Nearby there is a bright red heart, just like the petals on my flower
Turn the drawing back over. Do you remember what Whizzy the Wizard said? Good. Let's colour it in! As soon as you've finished, turn the drawing face down again and listen.
All the other petals are blue, but the colour of the centre is up to you!
The mushroom's cap is deep red, but the stork is bright blue!
Turn the drawing back over. Do you remember what Whizzy the Wizard said? Good. Let's colour it in! As soon as you've finished, turn the drawing face down again and listen.
At the top of the three there are three apples, one red, the other blue, and the last one's colour is up to you!
Turn the drawing back over. Do you remember what Whizzy the Wizard said? Good. Let's colour it in! As soon as you've finished, turn the drawing face down again and listen.

The tree trunk has four horizontal stripes, one red, one yellow, one green and the last one is blue!
Turn the drawing back over. Do you remember what Whizzy the Wizard said? Good. Let's colour it in! As soon as you've finished, turn the drawing face down again and listen.
Little elves, you've been so good. You're almost finished, but listen to what I say next:
Before you can ask me for the Potion, you must colour is the tree's leaves. I want them to be equal parts red, yellow and blue. But if they're uneven, you can't come through!
Well done! You've passed the test!

THANKS TO THE ACTIVITY THE CHILDREN.…
They practise paying careful attention to outside information and they will practise checking their answers, while still playing by the rules of the game. As well as controlling their impulses, this activity reinforces their working memory.
Thinking back on the game…
When the children have to fetch the drawing and the colouring markers, they have to be able to control their motor responses based on the words that they hear and the rules of the game.
Changing the rules of the game helps them to train their cognitive flexibility, that is to say know how to change their actions when necessary.
When the children have to listen to Whizzy the Wizard's colouring instructions the children learn to filter the important information as well as remember the instructions and suppress the desire to colour in the drawing immediately.
During this increasingly complex activity, the children are stimulated to make themselves think before they act.

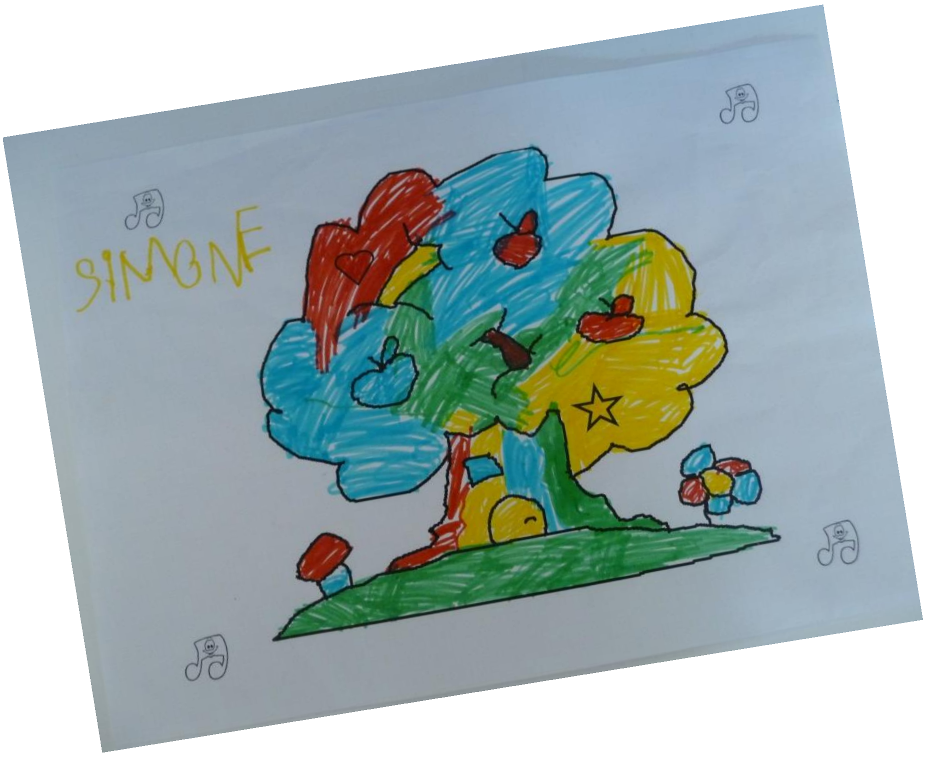

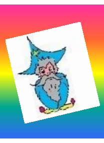

6TH MAGIC TEST
Chucky, Nelly
Looking for Whizzy the Wizard

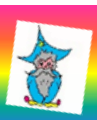

MATERIALS

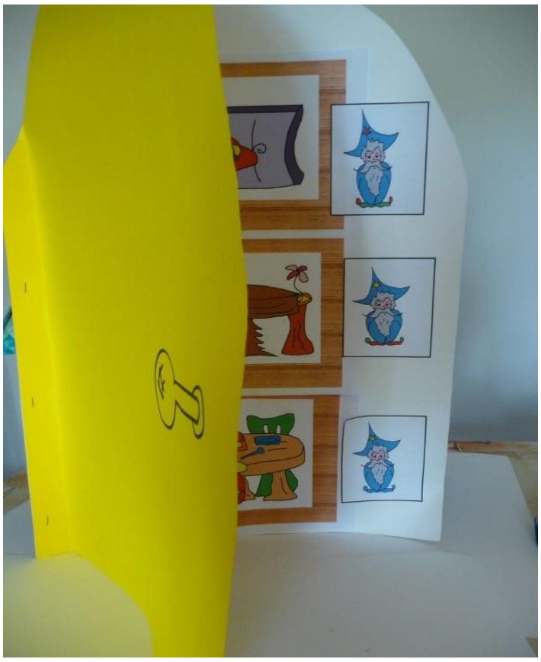

- ✓ Using a piece of cardboard, cut out a door that's big enough to fit in the slides 4, 5 and 6. Glue them next to the Whizzy the Wizard's house (slides 1-2-3)
- ✓ Make a small envelope for each child by folding a piece of cardboard in half. It must be big enough it fit the cut outs from slide 7-21.
- ✓ Make the same envelope for the teacher, but make it a different colour.
- ✓ Print outs of the self-assessment chart (slide 22, one for each participant) and some felt tipped pens .

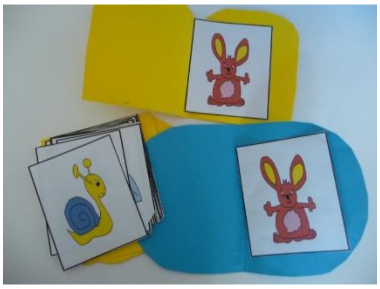

STRUCTURE OF THE ACTIVITY

Beginning

Hello everyone!

Come sit in a circle! (use place cards for each student until they are able to find their place on their own).

Do you remember why we're here? …. (help the kids answer) Yes! We're here to help Chucky and Nelly.

What have we done so far? … Yes! We got to Whizzy the Wizard's house!

Do you know what we're going to do now? We have to pass the sixth magic test!

But first: The Super Magic Attention Song! (sing and dance together)

Game

Do you know what the sixth magic test is? Today … (explain the game on the following paragraphs).

Ending

You've been so good, we've passed the sixth magic test! Now we're each going to take this drawing (slide 30) and choose three colours. (Once the children have chosen their three colours, tell them which colour corresponds to which state of attention.) While we're colouring in, tell me if you think we've past the test? (repeat what the children say, sharing any useful strategies e.g.: we kept quiet while we were listening, we whispered to ourselves whatever we had to remember…).

Have you finished colouring? Good! Everyone stand up, one hand above the other. Now say "We have found Whizzy the Wizard!" Yay!

THE GAME

In this game, each child has a picture of the same character but only two have the same character with the same details. The child with the secret character has to discover who has the same of him/her by questioning. The children have to look at their picture one time then they have to remember it. Finally, they have to listen at the Whizzy the Wizard's look and find him saying where he is.

THE GAME'S INSTRUCTION

Last time we coloured in Whizzy the Wizard's house. Today we're going to see if we can get in. Here is the door (show the kids the cut out door you prepared).

Do you remember that you coloured Whizzy the Wizard's door yellow? This is the same door! Let's knock. Whizzy the Wizzard? Whizzy the Wizzard, are you there? Try calling him. Look, if we crack open the door we can see the rooms of the house. Here's his bedroom, can you see his mushroom bed? Here is where he mixes his potions, and here is where he eats!

But have you seen how sly the wizard is? He's made many little copies of himself so it's hard for us to find the real one! They are all of these little wizards in these rooms.

But look, they're not al exactly the same! Some of them are a little different. For example, these ones have different coloured shoes. We have to find out which is the real Whizzy the Wizard! (close the door an put it away)

In order to find out who is the real wizard, we can practise with a game. The game is called "Hide all you want, mystery creature, I will find you! I'm the seeker!" Do you know it?

It works like this: I've got this blue door with the mystery creature inside (the snail). You have got the little yellow doors. Now everyone gets a picture of a snail (the page with the snail). Quickly put it inside your door and close it so that no one else can see inside. Now I will give you some clues so that you can find out who among you has the mystery creature that I have. Wait until I have given you all of the clues. Whoever thinks they have the mystery creature must keep quiet and pretend they don't know. Don't let anyone know if you have it, until I ask you to put your hand up if you think you have the mystery creature. You now have six seconds to look at your picture and memorise the colours. Then you must close the door. Have you memorised your picture? Good, now listen up…

The mystery creature moves slowly and carries his house on his back.
His body's yellow and his shell is blue!

Keep your doors closed. You can't open them up to see what your picture is. Whoever thinks they have the mystery creature I just described, put your hand up! Let's see. (The teacher checks to see if the picture is the same as the one they described.) Well done!

Now give me back the snails and I will give you another picture (butterfly). Look at your pictures. You now have six seconds to look at your picture and memorise the colours. Then you must close the door. Have you memorised your picture? Good, now listen up…

(butterfly) The next mystery creature has such beautiful wings
His body is green,
His wings are too,
But the outline of his wings are red.

Whoever thinks they have the mystery creature I just described, put your hand up! Let's see. Well done! Now give me back the snails and I will give you another picture (cat).

(cat) The next mystery creature has two ears and a long long tail.
His body's blue
And he's got a white heart on his tummy.
The outline of his ears is yellow.

Whoever thinks they have the mystery creature I just described, put your hand up! Let's see. Well done! Now give me back the snails and I will give you another picture (bee).

(bee) The next mystery creature has got black and yellow stripes like you've never seen!
His wings are blue,
His antennae too,
But his shoes are green.

Whoever thinks they have the mystery creature I just described, put your hand up! Let's see. Well done! Now give me back the snails and I will give you another picture (rabbit).

The next mystery creature has got two long ear and pink tummy
His nose is black and his ears are yellow

Whoever thinks they have the mystery creature I just described, put your hand up! Let's see. Well done, everyone! Now we've had enough practice to find the real wizard!

Let's open up the door. Look at all the wizards…

Now I'm going to give you some clues so that you can find the real wizard. You have to wait for me to give you all the clues, and then you can tell me which is the real wizard, and which room he's in.
Listen up:
You won't find Whizzy the Wizard today if you don't listen to everything I have to say, okay?
Whizzy's beard is grey and his robes are blue
His funny hat is pointy, oh! And it's blue, too!
On his has is the strangest star you've ever seen
It's strange because it isn't yellow, it's green!
The last thing you need to know about the wizard, my friends
Is that his shoes are yellow, with fluffy pink balls on the end!
Have you found the real wizard?
Good, now I'm going to close the door.
If you can tell me which room the wizard is in, then you've passed the test.
Well done! You've passed the test!

THANKS TO THIS ACTIVITY THE CHILDREN…
They strengthen their memory, especially their verbal and visuospatial memory (they have to remember verbal and visual information) and their inhibitory capacity
Thinking back on the game…
The most important capacity to pass the test is memory. The children have to be able to memorise the colours of the pictures, and not forget them while they listen to the teacher's clues. Moreover, in order to succeed, the children have to pay careful attention to what the teacher is saying. They also have to look carefully at the details of the pictures. If they get distracted, they won't know if their picture is the right one or not.
The entire game requires them to control their impulses. They must not look at the other students' pictures, nor must they say which picture that have. They cannot peek inside their own envelope to check their picture. And finally, they have to keep still until all the clues have been said, and until the teacher asks them to put their hand up.
The complexity of this game trains the children to pay attention to their surroundings, remember useful information for their goals, work with memorized information and suppress the impulse to respond immediately to the stimuli.

7TH MAGIC TEST
Whizzy the Wizard wants to know if Chucky and Nelly can control their actions!

MATERIALS

- ✓ Cut out the pictures on slides 2 and 3 and glue them onto coloured cardboard strips. Staple them into a ring, to make hats for the Time Elf, the Magic Water Elf, the Clapping Elf, and the Magic Potion Elf;
- ✓ Five plastic bottle lids in a plate
- ✓ Water
- ✓ A "Potion" bottle;
- ✓ A printout of the self-assessment chart (slide 7, one for each participant) and felt tipped pens
- ✓ A timer (a watch, an hourglass or a stopwatch)
- ✓ Cutouts of slides 4-5-6
- ✓ Paper tape to make the following diagram (see also slide 1):

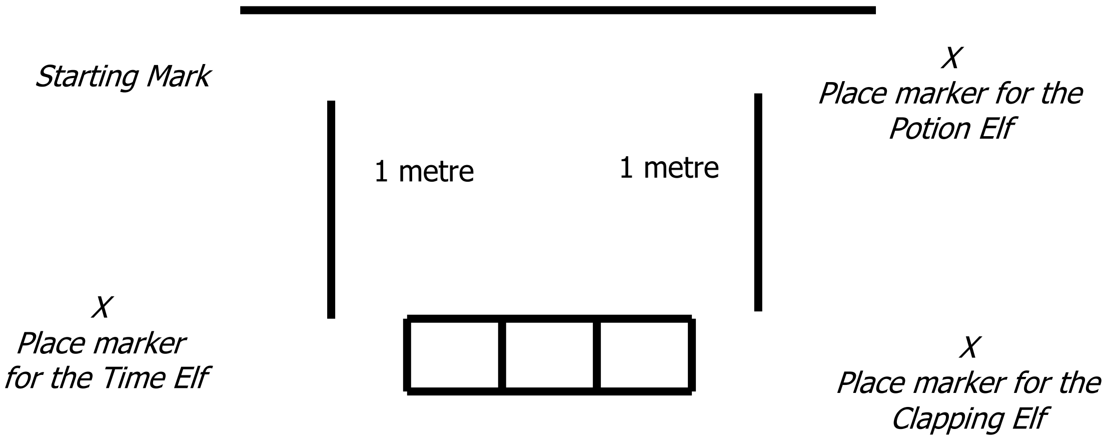

STRUCTURE OF THE ACTIVITY

Beginning

Hello everyone!

Come sit in a circle! (use place cards for each student until they are able to find their place on their own).

Do you remember why we're here? …. (help the kids answer) Yes! We're here to help Chucky and Nelly.

What have we done so far? … Yes! We got to Whizzy the Wizard's house!

Do you know what we're going to do now? We have to pass the seventh magic test!

But first: The Super Magic Attention Song! (sing and dance together)

Game

Do you know what the seventh magic test is? Today … (explain the game on the following paragraphs).

Ending

You've been so good, we've passed the seventh magic test! Now we're each going to take this drawing (slide 30) and choose three colours. (Once the children have chosen their three colours, tell them which colour corresponds to which state of attention.) While we're colouring in, tell me if you think we've past the test? (repeat what the children say, sharing any useful strategies e.g.: we kept quiet while we were listening, we whispered to ourselves whatever we had to remember…).

Have you finished colouring? Good! Everyone stand up, one hand above the other. Now say "Little Elves know how to pay attention and they can control their actions! We passed the test! Yay!"

THE GAME

The children have to perform an obstacle course. As usual each child is a players and then he/she has the role to ask to other children something and to control them.

THE GAME'S INSTRUCTION

Today we have to help Chucky and Nelly pass Whizzy the Wizard's first challenge!
Whizzy the Wizard will only give Chucky and Nelly the potion if they avoid distractions
The wizard says they have been so patients and they know how to control their actions!
Before we do the actual game, you have to be able to sit still and quiet on this waiting line for three minutes, no matter what I do or say. (Explain to the children that the time is up when the arrow gets to the number three, or use an hourglass).
(After the first three seconds, tease the children by asking them their name, what they like to eat or their favourite colour).
Well done! Now I will explain to you how to play the game.
To show Whizzy the Wizard that we can be very careful and we can control our movements we have to be able to do this obstacle course. We have to full up the potion bottle with Magic Water all the way up to here (show the line on the bottle).
Do you see this line (point to the tape on the floor, see slide 1). Each Elf Player must do this obstacle course, one at a time.
First of all, the Elf Player must balance on this line for at least twenty seconds.
The Time Elf (call a child) who is wearing a hat with a clock, must check to see that the Elf Player takes at least twenty seconds.
If the Elf Player gets to the end of the line after the Time Elf has finished couting to twenty, then the Time Elf can give the Elf Player a lid. If not, he must go back to the beginning.
Once the Elf Player has passed this line with the lid, he must move towards the Magic Water Elf, who is wearing the water hat and is sitting here next to these three squares.
I will stand at the end of these three squares. From here I will show the Elf Player some coloured cards and say some colours out loud. The Elf Player can move forward only if I say red while I'm showing a red card (cut outs from slides 4-5-6; say a colour and then show a coloured card, following Table 1, on slide 1)
If the Magic Water Elf says that the Elf Player did everything right, then he can carefully pour some Magic Water into the lid. The Elf Player with the lid full of Magic Water must be able to balance all the way to the Potion Bottle that the Potion Elf is holding.
Be careful! If the Clapping Elf, who is wearing this hat and is sitting here next to the line, claps his hands, the Elf Player must kneel down without letting the water spill.
Once he gets to the end of the line and the Potion Elf, the Elf Player pours his Magic Water into the Potion Bottle.
The children do the obstacle course, taking turns to be the different roles. (Encourage the children to be autonomous in playing the game, after helping them to remember the rules the first time round).
Well done! You have passed the test!

THANKS TO THIS ACTIVITY THE CHILDREN…

They exercise their inhibitory capacity.
Thinking back on the game…
In the first stage of the obstacle course the Elf Plyers have to be able to move slowly, then they have to suppress their impulse to jump, then they have to be able to move carefully so that they

don’t spill the water and bring it to the Potion Bottle. This continuous regulation of movements helps the children to exercise their inhibitory capacity.
During this game is it also necessary to remember the rules of the obstacle course. The Time Elf, the Magic Water Elf, the Clapping Elf and the Potion Elf have to continuously pay attention to what the Elf Player is doing. They have to be active in supervising the activity.

8TH MAGIC TEST
Whizzy the Wizard wants to know if Chucky and Nelly can pay attention!

MATERIALS

- ✓ One print out for each participant of slides 1,2,3,4,5,6
- ✓ Felt tipped pens

A printout of the Self-Assessment Chart
(slide 7, one per participant)

Die Elf Chucky and Nelly Elf Checker Elf Puzzle

Striscia di scotch di carta messa a terra

Teacher X X Elf Player

STRUCTURE OF THE ACTIVITY

Beginning

Hello everyone!
Come sit in a circle! (use place cards for each student until they are able to find their place on their own).
Do you remember why we're here? …. (help the kids answer) Yes! We're here to help Chucky and Nelly.
What have we done so far? … Yes! We did Whizzy the Wizzard's first challenge! We know how to control our actions!
Do you know what we're going to do now? We have to pass the eighth magic test!
But first: The Super Magic Attention Song! (sing and dance together)

Game

Do you know what the eighth magic test is? Today … (explain the game on the following paragraphs).

Ending

You've been so good, we've passed the eighth magic test! Now we're each going to take this drawing (slide 12) and choose three colours. (Once the children have chosen their three colours, tell them which colour corresponds to which state of attention.) While we're colouring in, tell me if you think we've past the test? (repeat what the children say, sharing any useful strategies e.g.: we kept quiet while we were listening, we whispered to ourselves whatever we had to remember…).
Have you finished colouring? Good! Everyone stand up, one hand above the other. Now say "Little Elves know how to pay attention! We passed the test! Yay!"

THE GAME

The children have to pay attention to visual or auditory stimuli to find what is requested.

THE GAME'S INSTRUCTION

Today Whizzy the Wizard wants to know if the Elves know how to pay attention.
Are you ready? Everyone needs a felt tipped pen (give a felt tipped pen to each child). Good. Now listen to what Whizzy the Wizard says (give each child a copy of slide 1).
Now that you have seen my hat (point to the hat at the top of the page) *you have twenty seconds to circle all the hats that are the same.* (Point to the hate at the top of the page and then point to the hats below, encouraging the children to circle the ones that look the same as the hat at the top. Count to twenty out loud). After twenty seconds ask the children to pass their sheet of paper to the Elf next to them so that they can check who found all the hats.
Well done! Now (Give each child a copy of slide 2) use the felt tipped pen to make a cross when you see a hat that's different from the one up top. (Point to the hate at the top of the page and then point to the hats below, encouraging the children to circle the ones that look the different from the hat at the top. Count to twenty out loud). . After twenty seconds ask the children to pass their sheet of paper to the Elf next to them so that they can check who found all the different hats. Well done! *Now* (Give each child a copy of slide 3) Whizzy the Wizard says: *On this sheet of paper you must find my magic wand! Circle all the wands that look the same as mine.* (point to the wand at the top and encourage the children to circle all the wands that look the same. Count out loud to twenty.)
After twenty seconds ask the children to pass their sheet of paper to the Elf next to them so that they can check who found all the wands. *Now* (Give each child a copy of slide 3) *you have to circle everything except my magic wand!* (point to the wand at the top and encourage the children to circle all the wands except the one that looks like the wizard's wand. Count out loud to twenty.)
*Well done! You've been so good at paying attention! But I still need to check your attention skills. Spot the differences between these two drawings (*give each child a copy of slide 4). *Can you spot the differences?*
You've done so well, you've managed to get to the last magic test of attention!
Now, you two little Elves must clap your hands each time you hear me say a piece of clothing, you two must each time you hear me say an animal, and the rest of use must clap our hands each time we hear food.
Are you ready? Listen to what Whizzy the Wizzard says:
When I went to the market do you know what I got?
I bought a giant ***jacket*** *to keep me hot*
I also bought some yellow ***stockings***
With mewling ***kittens*** *on each one*
I was tired from doing so much walking
I saw lay down for a bit in the sun
And then what was there before my eyes?
A great big ***dog****, to my surprise!*
And with the dog there was a ***gnat***
Who was wearing a little ***hat***
Then I stood up and went to find
Some food, that was on my mind
I had a bowl of ***soup****, so hot!*
And afterwards some ***chocolate****!*
Then I went to say hello
If you know to whom I'll let you go!
(Show the children the printout of slide 5) *At the market you'll fin Rose. Can you see she's wearing witch's clothes. Her table is big and brown. And yellow curtains all around. They are tied with bright red string. On her head is a very strange thing. A witch's hat, can you see? It's purple and it's got a wee little red heart. And I can see too, it's got a flower that's orange and blue. Her robes are green, her necklace is blue. And just like a tree, her hair's green, too!*
Do you know who it is? Well done!!! We've passed the test!

THANKS TO THE ACTIVITY THE CHILDREN…

The children exercise their attention skills

Thinking back on the game…

In the first part of the game the children have to be able to filter the visual input in order to find the drawing they need among many other drawings. Moreover, when they are asked to not circle the targeted stimulus, they have to modify the rule with which they were analysing the stimuli, thereby directive their attentive resources to a new objective.

During the activity, the children move from visual stimuli to auditory stimuli. When the children have to clap their hands depending on the stimulus in the wizard’s rhyme, they exercise their auditory attention.

Finally, in the last tsk the children exercise their attentive resources towards visual and auditory stimuli.

9TH MAGIC TEST
Whizzy the Wizard wants to know if Chucky and Nelly can remember details!

MATERIALS

- ✓ Printouts of slides 1-5 to be cut to make the cards for the game.
- ✓ Printouts of slides 1-5 to be the children's chart.
- ✓ 3 Dice
- ✓ Self-assessment chart (slide 6, one for each participant) e felt tipped pens

STRUCTURE OF THE ACTIVITY

Beginning

Hello everyone!

Come sit in a circle! (use place cards for each student until they are able to find their place on their own).

Do you remember why we're here? …. (help the kids answer) Yes! We're here to help Chucky and Nelly.

What have we done so far? … Yes! We showed Whizzy the Wizard that elves can pay attention!

Do you know what we're going to do now? We have to pass the second last magic test!

But first: The Super Magic Attention Song! (sing and dance together)

Game

Do you know what the ninth magic test is? Today … (explain the game on the following paragraphs).

Ending

You've been so good, we've passed the ninth magic test! Now we're each going to take this drawing (slide 11) and colour it in depending on how well you think you paid attention. Colour in the the smiling die if you think you paid attention very well. Colour in the straight-faced die if you think you paid attention, but it was a bit difficult. And colour in the sad die if you don't think you paid much attention. While we're colouring, how do you think we passed the test? (repeat what the children say, sharing any useful strategies e.g.: we kept quiet while we were listening, we whispered to ourselves whatever we had to remember…).

Have you finished colouring? Good! Everyone stand up, one hand above the other. Now say "We know how to think before we act! We have passed the test! Yay!"

THE GAME

The game consist on a magic memory game.

THE GAME'S INSTRUCTION

Today Whizzy the Wizard wants to know if Chucky and Nana are Elves who can remember all the elements!

While you're sitting in a circle, I'm going to give you each one of these charts (give each child one of the charts from the slides 1-2-3-4-5).

Now I'm going to put all these cards (the pictures that have been cut by 1-2-3-4-5 slides) face down on the table.

In order to pass this test you have to be able to find the same cards as the ones on your chart.

But we have to follow some rules!

The first Elf to play has to first of all throw these three dice and then say which of the dice has the highest number (the next turn he must say which is the lowest number, and then again which is the highest). If he's right, he can then turn over as many cards as the highest number.
If one of the cards he turns over is one of the ones he's looking for, before he can take it he must put his hands on the ground and say Memory Master!
The last rule is that you have ten seconds to look at your charts now and memorise which cards you are looking for. Once you've memorised it, you must turn your chart face down. Try to remember your cards while you're playing. You can look at them only when it's your turn.
In order to pass the test, everyone has to fill out their chart.
Are you ready? Let's begin!
Well done! We have finished all the cards! Let's see if we've all been able to fill out our charts. If we have, then we've passed the test!
Well done!!
You are all Memory Masters! We've passed the test!

THANKS TO THIS ACTIVITY THE CHILDREN….
They exercise their memory most of all.
Thinking back on the game…
As well as having to remember which pictures to look for, the children have to use their mnestic resources to compare the dice numbers and remember what to do and say when they want to get the card they need.

10TH MAGIC TEST
Whizzy the Wizard has finally given the children the super magic attention potion!

MATERIALS

✓ Cut outs of slide 1, stuck on to long strips of coloured cardboard that can be stapled to make a hat. One hat for the Die Elf, one for the Puzzle Piece Elf, one for the Checker Elf, and one for the Chucky and Nelly Elf.
✓ 3 dice in a plate.
✓ 1 20-piece puzzle (see slide 5) and two plastic containers to put them in.
✓ Paper (cut outs of slides 2-3-4)
✓ Self-assessment chart (slide 6, one for each participant) and felt tipped pens
✓ 1 piece of paper tape to make a 1,5 metre line and to make place markers.

STRUCTURE OF THE ACTIVITY
Beginning
Hello everyone!
Come sit in a circle! (use place cards for each student until they are able to find their place on their own).
Do you remember why we're here? …. (help the kids answer) Yes! We're here to help Chucky and Nelly.
What have we done so far? … Yes! We showed Whizzy the Wizard that we can remember details!
Do you know what we're going to do now? We have to pass the last magic test!
But first: The Super Magic Attention Song! (sing and dance together)
Game
Do you know what the last magic test is? Today … (explain the game on the following paragraphs).
Ending
You've been so good, we've passed the last magic test! Now we're each going to take this drawing (slide 12) and choose three colours to colour it in depending on how well you think you paid attention. (One the children have chosen their three colours, tell them which colour corresponds to having paid attention, which corresponds to having found it difficult to pay attention, and which corresponds to not having paid attention.) While we're colouring, how do you think we passed the test? (repeat what the children say, sharing any useful strategies e.g.: we kept quiet while we were listening, we whispered to ourselves whatever we had to remember…).
Have you finished colouring? Good! Everyone stand up, one hand above the other. Now say "We know how to think before we act! We have passed the test! Yay!"

THE GAME
The children have to win the puzzle pieces and then make the puzzle all together.

THE GAME'S INSTRUCTION
Listen to what Whizzy the Wizard has to say:
Well done, little elves, you've paid attention!
In order to get the Magic Potion you have to pass the last test. You little elves know that you have to think before you act!
Whizzy the Wizard wants to see if we can do this puzzle (show the children the drawing). But to do it we first have to win all the puzzle pieces (show the children the loose puzzle pieces).
Now I'll explain to you how it works.
Sit down on the line (point to the tape line on the floor).
(Assign to each child one of the roles and give them the appropriate materials).
The Die Elf, who's wearing this hay, have to these three dice in this plate.
The Chucky and Nelly Elf must wear this hat and must hold these two cards, one with Chucky and the other with Nelly.

The Puzzle Piece Elf must wear this hat and has to do a very important task: he has to hold this container with all the puzzle pieces. He must be very careful not to drop them.
The Checker Elf must wear this has and must look to see that the Elf Player is doing everything right.
The Elf Player must stand in front of me.
First, the Elf Player must go to the Die Elf and throw the three dice. He must then say which is the highest number. He must remember this number all the way to the end of his test. The Die Elf must also remember the number, so that and the end of the test he can check to see if the Elf Player got it right.
Then the Elf Player must go to Chucky and Nelly Elf. The Chucky and Nelly Elf must decide whether to give the Elf Player the card with Chucky or the card with Nelly.
Then the Elf Player must come stand in front of me.
When I say "Cat" and you see a cat (show them the cut outs from the slide) you must jump.
When I say "Mouse" and you see a mouse (show them the cut outs from the slide) you have to touch the ground. If I say "Cat" but you see a mouse you have to stay very still!
One last rule: If you see Chucky or Nelly, depending on which card the Chucky and Nelly Elf gave you, you have to do the right action (jump up or touch the ground) but you must also say "Chucky" or "Nelly" (During the game, show the cards one at a time. Show different pictures three times, e.g.: a can and a mouse)
The Checker Elf must look and see the Elf Player does everything right. Once he has checked, the Elf Player must say his number out loud. If the Die Elf says it's the right number, then the Elf Player has passed the test and he can go collect four puzzle pieces from the Puzzle Piece Elf. Put the puzzle pieces in the plate. Well done! (Help the children to understand the game and to oversee the game themselves).
Now we're all going to swap places. The Elf Player must take the Die Elf's place, the Die Elf must take the … (do two rounds)
We've managed to get at least ten puzzle pieces! We've passed the test, and now we can do the puzzle! Let's see (Count the puzzle pieces with the children).
Well done!
Now let's sit in a circle. We must do the puzzle. Remember that elves think before they act, so before we begin we should look at the picture (slide 5). Now I'm going to give you each four puzzle pieces. You have to complete the puzzle by working together.
You've got forty seconds to do it! Ready, set, go! (Count to forty out loud)
Well done! We've passed the test!

THANKS TO THIS ACTIVITY
They practise their capacity to think ahead, to think before they act.
Thinking back on the game…
During the first part of the game, the children have to change their own actions in a short amount of time, depending on what stimulus they receive and the rules of the game.
During the puzzle activity, the children have to be able to work as a group and at the same time figure out where to put their own puzzle pieces.

THE END
Finally Chucky and Nelly are elves again, thanks to the children's help!

MATERIALS

- ✓ Print outs of slide 1 (one for each participant)
- ✓ Cut out the clothes in slide 2 (one copy for each participant)
- ✓ A 'microphone' (a pen);
- ✓ A cape made from a piece of material;
- ✓ A box with two holes that can be used as a camera
- ✓ A piece of cardboard that has been folded in half, small enough to fit in a pocket, into which you must glue a piece of reflective cardboard, like a mirror;
- ✓ Print out of the slide 3 (one per participant);
- ✓ Felt tipped pens;
- ✓ A stuffed bird (Harry the Hummingbird).

THE GAME

The children have to perform an obstacle course and to find out the Chucky and Nelly's clothes.

THE GAME'S ISTRUCTION

Fantastic! We have passed all ten magic tests!
Chucky and Nelly are so happy that they told me to thank you!
But they asked me if you could be so kind as to help them find their elf clothes.
Look here, this is the obstacle course we must do, the pen and these are their clothes, (give each child a print out of slide 1, the cards cut out from slide 2 and a felt tipped pen), and listen to what Nelly has to say:
Hello friends!
Thank you so much for helping us.
Now can you help us find our elf clothes.
You'll find them in the labyrinth, but first you have to know what to look for!
Chucky has a pointy blue hat with a yellow star!
My hat is yellow with a bright pink heart,
My dress is pink with a yellow trim
And oh! I forgot my shoes are pink too!
Chucky's shoes are green, his top is yellow and his pants are blue!
(Ask the children if they've figured out which clothes are elves')
Now you are ready to go find the clothes in the labyrinth!
Good! Well done!
(Surprise the children by suddenly bringing out Harry the Hummingbird from behind your back)
Do you remember Harry the Hummingbird? He came to tell me that you have become famous! Chucky and Nelly have told all of their friends that you were so good at helping them. Whizzy the Wizard has said we must have an interview on television!
You can be the journalist (put the cape on the child), you can hold the microphone, you can hold the camera, and you can give the Potion recipe and this magic card. Everyone is going to get the chance to have an interview.
The journalist must remember to ask:
1. If he was happy to help Chucky and Nelly
2. What helped to be so good at helping Chucky and Nelly.
3. If he wants this magic card (a card that has been prepared in advance).
(encourage the children to take turns in the different roles)
Well done! Now I'm going to tell you what this Magic Card is for. Anytime that we're having a bit of trouble paying attention or doing what we want, we're going to open the card, look at ourselves

in the mirror and say Super Magic Attention Potion and it will transform us into Super Attentive Children, who know to think before they act.
Now put that card in your pocket right away! It's secret present from Chucky and Nelly.
Now I'm going to give you this drawing (slide 7) that you can colour in however you wish. You can also draw Chucky and Nelly and put these clothes on them (cut outs from slide 6) if you want!
You've done so well!
Now everyone stand up, put one hand above the other, lift them up and say "Chucky and Nelly are elves again!"
Everybody clap!
Thank you for your help!

THANKS TO THIS ACTIVITY THE CHILDREN…
The children train their control of impulse, their attention, their memory, their flexibility and their planning. They increase their self-esteem and they think they are good in self-regulation!!!
